# Thermo‐Responsive Tri‐State Photonic Crystals

**DOI:** 10.1002/advs.202506507

**Published:** 2025-06-04

**Authors:** Yuewei Zheng, Jinqing Chen, Wanqi Mo, Wei Hong

**Affiliations:** ^1^ Key Laboratory for Polymeric Composite and Functional Materials of Ministry of Education School of Chemistry Sun Yat‐sen University Guangzhou 510275 P. R. China

**Keywords:** organic afterglow, photonic crystals, structural colors, thermochromic, tri‐mode encryption

## Abstract

Counterfeiting remains a pervasive global challenge, persistently undermining legitimate enterprises. Despite advancements, current anti‐counterfeiting materials and technologies fall short in addressing the escalating sophistication of counterfeit activities. A significant hurdle in this domain is the difficulty in achieving multi‐mode dynamic anti‐counterfeiting materials. Herein, thermo‐responsive tri‐state photonic crystals is presented that enable thermochromism of structural color, fluorescence (FL), and organic afterglow. The fabrication of this system integrates the incorporation of organic phosphors into polymeric colloids via solid‐phase extraction and embeds thermochromic microcapsules into colloidal crystals to regulate incoherent scattering and far‐field light absorption. In typical triplet‐to‐singlet Förster resonance energy transfer (TS‐FRET) for afterglow color tuning, higher energy transfer efficiency reduces the afterglow lifetime. In contrast, the far‐field light absorption‐based energy transfer in this photonic crystal system avoids this trade‐off, thereby preserving the long‐lived triplet emission. The resultant photonic crystals exhibit three thermochromic optical states including FL, phosphorescent, and structural coloration. This general strategy, leveraging tunable thermochromic temperatures and diverse organic phosphors, enables the creation of multi‐responsive anti‐counterfeiting patterns. It paves the way for dynamic, high‐capacity, and multimodal information storage.

## Graphical abstract

Tri‐state photonic crystals combine thermochromic structural color, fluorescence, and afterglow via far‐field absorption tuning, bypassing the efficiency‐lifetime trade‐off of TS‐FRET. This enables dynamic, high‐capacity anti‐counterfeiting with tunable optical states.

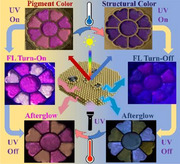

## 1 Introduction

The rapid advancement of technology has fueled the spread of counterfeit goods and information security threats, endangering product authentication, public health, and national security. To address these challenges, significant efforts have been devoted to developing advanced information storage systems and anti‐counterfeiting materials, ensuring secure data preservation and enhanced anti‐counterfeiting protection.^[^ ^1^ ^]^ Among various strategies, the development of multi‐modal encryption techniques has proven particularly effective for enhancing information security, which are capable of displaying distinct static information patterns under varying viewing conditions. To achieve such multi‐modal anti‐counterfeiting capabilities, it is effective to integrate dual or multiple optical functional components into a single anti‐counterfeiting material. To date, numerous multi‐modal anti‐counterfeiting systems have been successfully developed, incorporating various combinations of optical properties. These include systems that merge structural coloration with fluorescent effects,^[^ ^2^ ^]^ integrate fluorescent patterns with surface microstructural features (such as wrinkles),^[^ ^3^ ^]^ and combine liquid crystals and fluorescent properties.^[^ ^4^ ^]^

Recently, extensive research has focused on combining physical and chemical structures to engineer materials exhibiting both structural and fluorescent colors.^[^ ^5^ ^]^ Notably, the luminescence intensity of the system can be significantly enhanced by the Purcell effect under specific conditions.^[^ ^6^ ^]^ On the other hand, although organic phosphorescence materials exhibit outstanding time‐dependent and stimuli‐responsive afterglow properties,^[^ ^7^ ^]^ there are almost no examples of pure organic long‐afterglow materials being combined with photonic crystals. Furthermore, it can be predicted that if responsive structural colors could simultaneously induce changes in fluorescence and afterglow color, multiple optical modes for information security and anti‐counterfeiting technologies could be achieved.

**Scheme 1:**
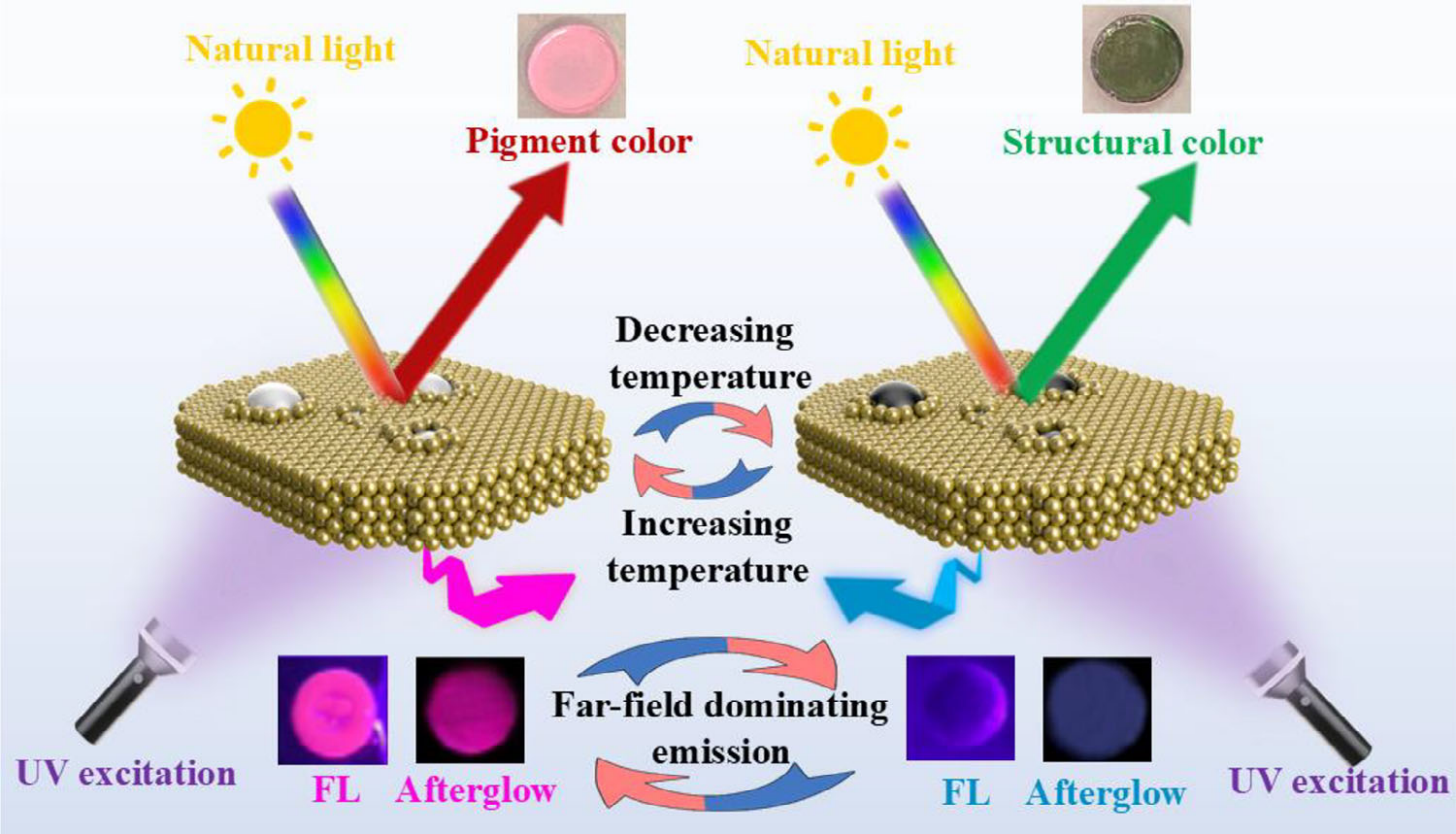
Schematic illustration of thermo‐responsive tri‐state photonic crystals enabling thermochromic switching between structural color, fluorescence (FL), and organic afterglow.

In this work, we demonstrate thermo‐responsive tri‐state photonic crystals that exhibit thermochromic fluorescence (FL), afterglow, and structural colors. The approach is based on two key hypotheses: (i) the incorporation of organic phosphors and dyes into polymeric colloids through solid‐phase extraction, and (ii) the incorporation of thermochromic microcapsules (MCs) to control incoherent scattering of the colloidal crystals and far‐field light absorption of the dyes (**Scheme** **1**). The resulting composite film exhibits three thermo‐responsive optical states: iridescent structural color and whitened appearance, thermochromic FL color, and thermochromic afterglow color. These multifunctional optical behaviors provide more complex optical information than traditional structural color materials and FL materials relying solely on either iridescent color or FL signals. The findings underscore the potential of multi‐modal anti‐counterfeiting systems based on photonic crystals and organic phosphorescence, enabling the creation of superparticle building blocks for advanced optical applications.

## 2 Results and Discussion

### 2.1 Synthesis and Luminescence Characteristics of ACD‐Me@NPs

**Figure 1:**
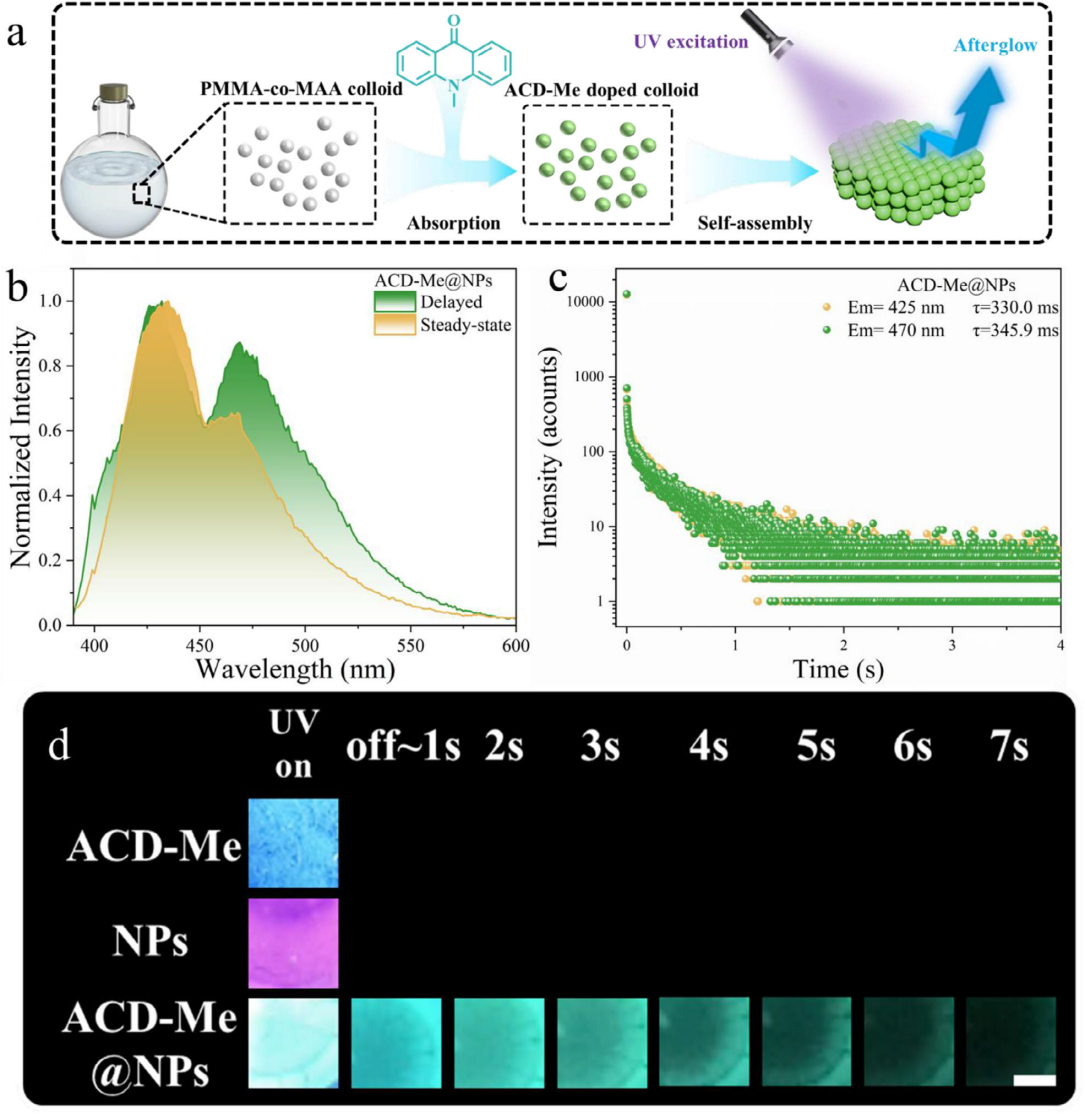
a) Schematic illustration of the preparation of ACD‐Me@NPs arrays, showing the molecular structure of ACD‐Me as the luminophore. b) Steady‐state and delayed photoluminescence (PL) spectra of ACD‐Me@NPs. c) Lifetime decay profiles of the delayed emission bands at 425 nm and 470 nm for ACD‐Me@NPs. d) Digital images of ACD‐Me powder, bare NPs, and ACD‐Me@NPs with UV irradiation on and off (λ_ex_ = 395 nm). (The violet color of the NPs originates from the excitation light.) Scale bar: 0.25 cm.

Submicron‐scale particles (NPs) were prepared via soap‐free emulsion polymerization of methylmethacrylate (MMA) and methacrylic acid (MAA), yielding an aqueous emulsion stabilized by the negative charge of sulfate ester and carboxyl groups on the particle surface (Scheme S1, Supporting Information). Upon addition of N‐methyl‐9‐ acridone (ACD‐Me) powder to the NP emulsion, the slightly soluble ACD‐Me was effectively absorbed into the hydrophobic interior of the NPs (**Figure** **1**a), forming ACD‐Me‐doped NPs (ACD‐Me@NPs) with a doping concentration of ≈0.01 wt.%. The UV–visible absorption spectra confirm the successful doping of ACD‐Me into the NPs rather than physical mixing (Figure S1, Supporting Information). After drying, the doped NP assembly exhibited intense blue FL peaking at 425 nm under 395 nm UV irradiation, as well as a long afterglow emission that was observed exclusively in ACD‐Me@NPs but not in pristine ACD‐Me or bare NPs (Figure 1d). The delayed emission spectrum displayed two distinct bands at 425 nm and 470 nm (Figure 1b).^[^ ^8^ ^]^ The 425 nm delayed emission band intensifies with rising temperatures (78–338 K), while the 470 nm band declines (Figures S2a–c, Supporting Information), indicating delayed fluorescence (DF) at 425 nm and room‐temperature phosphorescence (RTP) at 470 nm. Additionally, the increasing proportion of delayed components with temperature (Figures S2d‐‐f, Supporting Information) further supports the DF nature. Notably, the S_1_ energy level is measured at 3.18 eV (based on the blue edge of the DF band at 338 K in Figure S2a, Supporting Information) in agreement with the time‐dependent density functional theory (TD‐DFT) simulation results (Table S1, Supporting Information), while the T energy level, measured at 2.76 eV (based on the blue edge of the RTP band at 78 K in Figure S2a, Supporting Information), can be ascribed to the T_2_ state. The simultaneous presence of RTP and DF can be attributed to the following key factors: i) The spin‐orbit coupling matrix elements (SOCME) between S_1_‐T_2_ and S_0_‐T_2_ are significantly larger than those of other adjacent T energy levels (Figure S3, Supporting Information). ii) The energy gap between T_2_ and T_1_ is sufficiently large (0.37 eV) and the polymeric framework is rigid, suppressing the non‐radiative relaxation from T_2_ to T_1_. iii) The substantial energy gap between S_1_ and T_2_ (0.54 eV) prevents a rapid reverse intersystem crossing (RISC) process. As a result, long‐lived DF emission is achieved (Figure 1c), along with RTP originating from the T_2_ energy level. The system exhibited a photoluminescence quantum yield of 75.8% and achieved an afterglow efficiency of 47.8% (Figure S4, Supporting Information). The method demonstrated universal applicability to various organic afterglow molecules that are slightly soluble in water, as evidenced by the successful doping of indolo‐[2,3‐a]‐carbazole (CAB) and 9H‐pyrido‐[3,4‐b]‐indole (IND) into NPs, which produced corresponding persistent luminescence (Figures S5 and S6, Supporting Information).

### 2.2 Energy Transfer‐Mediated Multicolor Afterglow in Dyes‐Co‐Doped NPs

**Figure 2:**
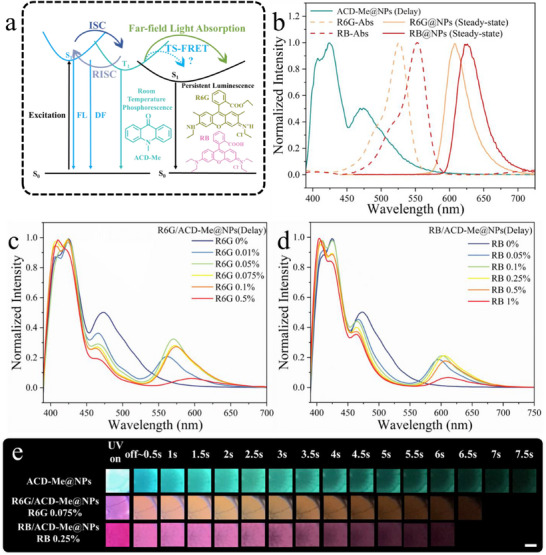
a) Jablonski diagrams of ACD‐Me@NPs, R6G/ACD‐Me@NPs, and RB/ACD‐Me@NPs. b) Delayed photoluminescence (PL) spectrum of ACD‐Me@NPs (bluish‐green curve), absorbance spectra of R6G (dashed yellow curve) and RB (dashed red curve), and PL spectra of R6G@NPs (yellow curve) and RB@NPs (red curve). c) Delayed PL spectra of R6G/ACD‐Me@NPs with varying R6G doping concentrations. d) Delayed PL spectra of RB/ACD‐Me@NPs with varying RB doping concentrations. e) Digital images showing luminescence of ACD‐Me@NPs, R6G/ACD‐Me@NPs, and RB/ACD‐Me@NPs under UV excitation (λex  = 395 nm) and in the dark, with R6G and RB doping concentrations of 0.075 wt% and 0.25 wt%, respectively. Scale bar: 0.25 cm.

To tune the afterglow color, R6G and RB were further doped into the system, respectively. The optical absorption profiles of R6G and RB show significant spectral overlap with the delayed emission spectra of ACD‐Me@NPs in the 450–550 nm range (**Figure** **2**b; Figure S7, Supporting Information), suggesting potential energy transfer between ACD‐Me and the co‐embedded acceptor dyes (Figure 2a). Despite the ionic and hydrophilic (relative to ACD‐Me) nature of R6G and RB, these molecules were firmly absorbed into the NPs, as demonstrated by centrifugation results (Figure S8, Supporting Information). Both R6G/ACD‐Me@NPs and RB/ACD‐Me@NPs exhibit characteristic delayed emissions at 570 and 600 nm, respectively, corresponding to the photoluminescence signatures of R6G and RB (Figure 2c,d; Figure S9, Supporting Information).

Figure 2c reveals a direct correlation between R6G concentration (0‐0.5 wt% in NPs) and the I_R6G_/I_ACD‐Me_ intensity ratio (570 nm vs 470 nm emissions), which is similar to Figure 2d for RB. Optimal performance was achieved at 0.075 wt% R6G and 0.25 wt% RB loading, where maximum 570/470 nm and 600/470 intensity ratios were attained before concentration quenching occurred (Figure S10, Supporting Information). Notably, the delayed emission lifetime remained stable with increasing R6G concentration (Figure S11, Supporting Information), except for the highest concentration (0.5 wt%) showing quenched emission. This observation suggests that the energy transfer mechanism is not dominated by near‐field triplet‐to‐singlet Förster resonance energy transfer (TS‐FRET) between ACD‐Me and R6G.^[^ ^9^ ^]^ Typically, the TS‐FRET mechanism operates via dipole‐dipole coupling, enabling non‐radiative energy migration. This process necessitates close spatial proximity between the molecular orbitals of the donor and acceptor, typically requiring a separation distance of less than 10 nm.^[^ ^10^ ^]^ Furthermore, the TADF band intensity did not follow the RTP band reduction (Figure 2c,d), even though both emissions originate from the same triplet excitons. This further supports our conclusion that far‐field light absorption, rather than near‐field TS‐FRET, governs the energy transfer process. This phenomenon can be attributed to the spatial separation between cationic dyes (R6G/RB adsorbed on the anionic NP surface) and ACD‐Me molecules (distributed within the hydrophobic interior), which prevents the close proximity required for TS‐FRET. (In fact, the incorporation of ACD‐Me into the NPs occurs much more slowly than that of R6G/RB, as demonstrated in Figure S12 (Supporting Information). Furthermore, drop‐casting an ACD‐Me solution onto the NPs array resulted in significantly weaker delayed emission compared to the pre‐formed ACD‐Me@NPs array as shown in Figure S13 (Supporting Information). In contrast, drop‐casting an R6G solution onto the ACD‐Me@NPs array produced delayed emission similar to that of the R6G/ACD‐Me@NPs array. These observations confirm that the ionic dyes R6G/RB adsorb primarily on the NPs surface, while the hydrophobic ACD‐Me is incorporated into the NPs interior.) Despite this spatial constraint, the doped NP system achieves tunable delayed emissions at 470, 570, and 600 nm due to effective far‐field light absorption, enabling multicolor afterglow output (Figure 2e). In addition, typical TS‐FRET involves a trade‐off between energy transfer and long‐lived afterglow. The higher the energy transfer efficiency, the shorter the afterglow lifetime becomes.^[^ ^9^, ^10^ ^]^ However, far‐field light absorption behaves differently—there is no direct trade‐off relationship between energy transfer efficiency and afterglow lifetime, which is beneficial for maintaining the long‐lived characteristic of triplet state luminescence (despite a slight decay in afterglow duration, attributable to the inevitable reduction in initial luminescence intensity caused by diminished dye absorption).

### 2.3 Thermochromic Regulation of Afterglow via Far‐Field Absorption Control

**Figure 3:**
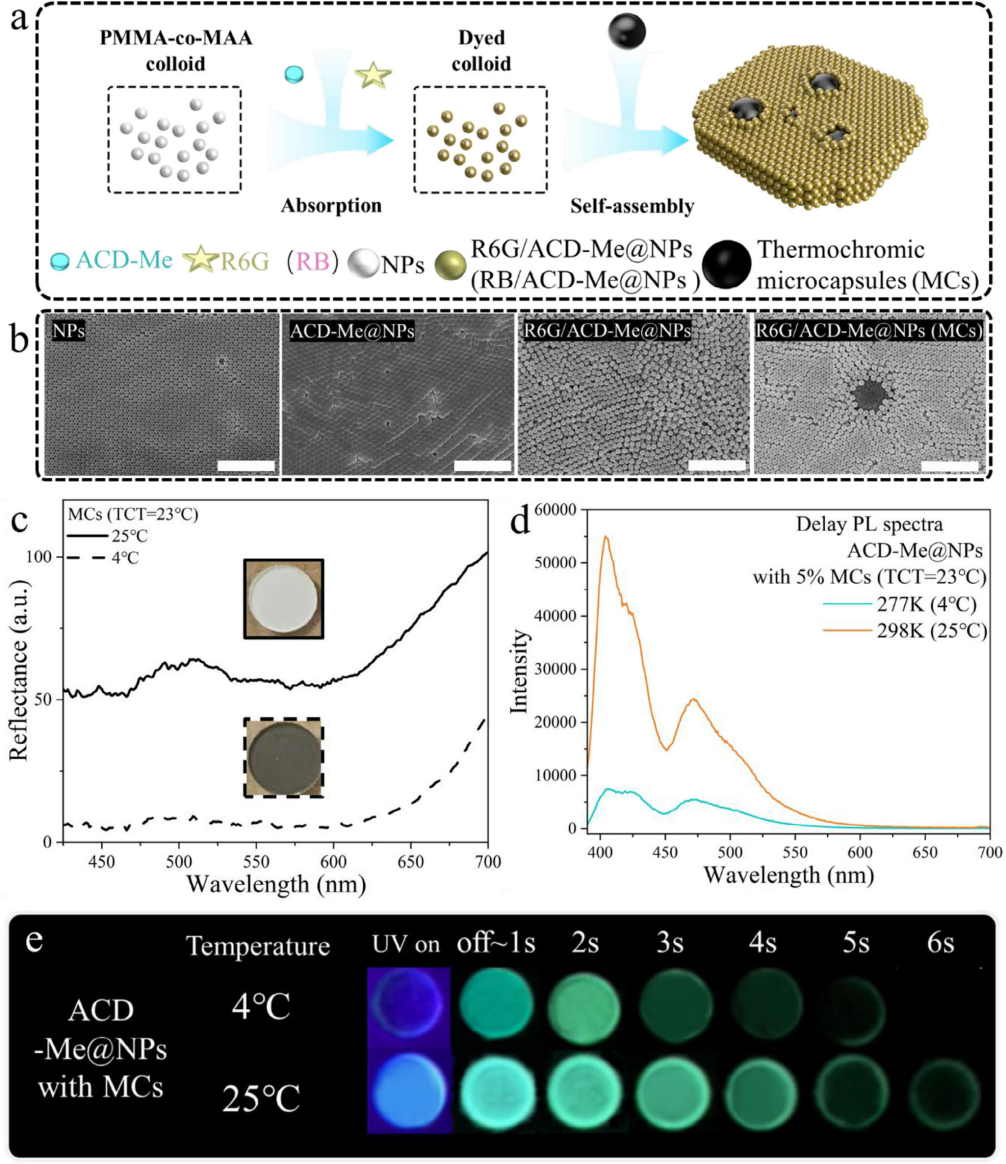
a) Schematic illustration of the preparation of thermo‐responsive tri‐state photonic crystals. b) SEM images of bare NPs, ACD‐Me@NPs, R6G/ACD‐Me@NPs, and R6G/ACD‐Me@NPs with MCs (scale bars: 2 µm). c) Reflectance spectra of MCs at 4 and 25 °C, with corresponding digital images shown above each temperature curve. d) Delayed PL spectra of ACD‐Me@NPs (with 5% MCs) at 4 and 25 °C. e) Digital images showing luminescence of ACD‐Me@NPs (with 5% MCs) under UV excitation (λ_ex_ = 395 nm) and in the dark at 4 and 25 °C.

Furthermore, responsive absorbers of thermochromic MCs were incorporated into the system via co‐assembly with the dyed NPs (**Figure** **3**a,b) to regulate far‐field light absorption. This system achieves broadband visible‐spectrum absorption through ODB‐2(2‐anilino‐6‐dibutylamino‐3‐methylfluoran)’s reversible lactone ring‐opening mechanism. Below the solid solvent's melting point (5–65 °C, tunable by solvent selection), MCs turn black due to phase‐transition behavior,^[^ ^11^, ^12^ ^]^ effectively suppressing far‐field light absorption. As shown in Figure 3c, MCs with a thermochromic transition temperature (TCT) of 23 °C exhibit reversible thermochromic behavior: the scattering background diminishes significantly below 20 °C, resulting in a dark grey appearance at temperatures <20 °C and a white appearance above 24 °C. Additionally, the FL and delayed emission of ACD‐Me@NPs were suppressed at low temperatures due to far‐field light absorption by MCs (Figure 3d). Although the lifetime of ACD‐Me@NPs slightly increased owing to stabilized triplet excitons at low temperatures (Figure S14, Supporting Information), the overall emission duration of the system with MCs decreased because of reduced emission intensity (Figure 3e).

**Figure 4:**
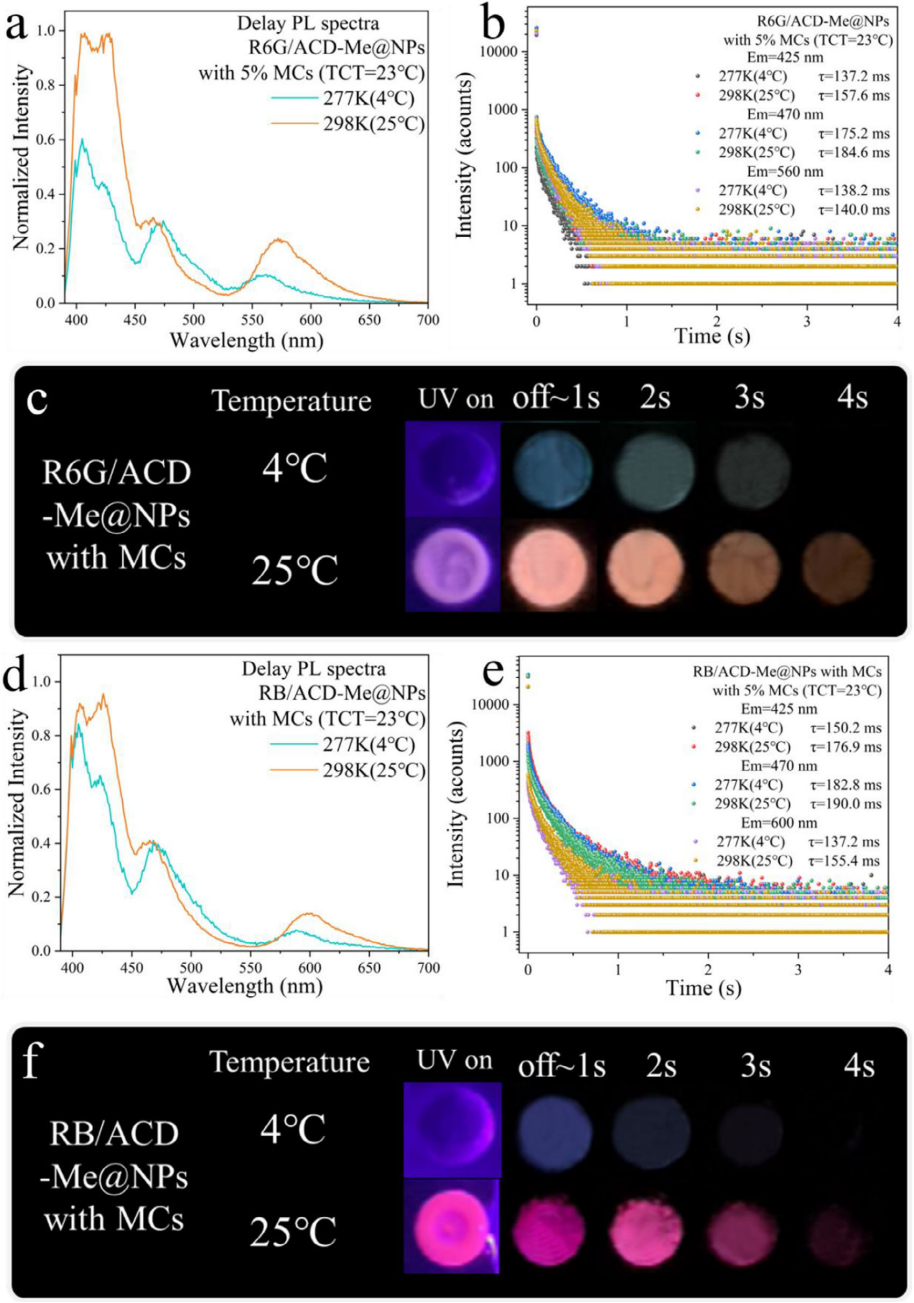
a) Delayed PL spectra of R6G/ACD‐Me@NPs (with 5% MCs) at 4 and 25 °C, normalized at 470 nm. b) Lifetime profiles of R6G/ACD‐Me@NPs (with 5% MCs) monitored at 425, 470 and 560 nm at 4 °C and 25 °C. c) Digital images showing FL and the afterglow of R6G/ACD‐Me@NPs (with 5% MCs) at 4 °C and 25 °C with UV excitation on and off. d) Delayed PL spectra of RB/ACD‐Me@NPs (with 5% MCs) at 4 °C and 25 °C, normalized at 470 nm. e) Lifetime profiles of RB/ACD‐Me@NPs (with 5% MCs) monitored at 425, 470 and 600 nm at 4 °C and 25 °C. f) Digital images showing FL and afterglow of RB/ACD‐Me@NPs (with 5% MCs) at 4 °C and 25 °C with UV excitation on and off. λ_ex_ = 395 nm.

Notably, the apparent energy transfer between ACD‐Me and R6G/RB was significantly suppressed by the MCs (**Figure** **4**). As shown in the delayed emission spectra normalized at 470 nm (Figure 4a,d), the characteristic delayed emissions of R6G (570 nm) and RB (600 nm) are effectively suppressed, although their corresponding lifetimes show limited changes (Figure 4b,e). This suppression manifested in distinct color changes under temperature variation: the delayed emission of R6G/ACD‐Me@NPs shifted from orange to steel blue when the temperature decreased from 25 to 4 °C (Figure 4c). Similarly, RB/ACD‐Me@NPs exhibited a chromatic transition from purple to dark blue‐purple (approaching deep Koamaru) under the same cooling conditions (Figure 4f). In addition, the enhanced intensity of the 470 nm RTP band relative to the DF band at 277 K reduced the spectral effects from the DF band tail, resulting in a red‐shifted RTP peak (Figure S15, Supporting Information). Meanwhile, the R6G (570 nm) and RB (600 nm) emission peaks blue‐shifted due to their diminished relative intensity and interference from the RTP band tail (Figure S15, Supporting Information), likewise contributing to a green‐toned afterglow. These synergistic shifts collectively produced the pronounced cool‐toned emission at 277 K observed in both R6G/ACD‐Me@NPs and RB/ACD‐Me@NPs. Interestingly, the photonic crystal structure was demonstrated to be essential for enabling thermochromic transitions in the afterglow color (Figure S16, Supporting Information). This phenomenon was absent in R6G/ACD‐Me‐doped transparent PMMA resin, which showed no thermochromic afterglow behavior. We attribute this distinction to enhanced far‐field absorption in photonic crystal arrays with strong scattering properties. Specifically, the photonic crystals significantly amplified long‐wavelength absorption by the MCs due to the decreased average photon mean free path (Tables S2 and S3, Supporting Information). Consequently, the RTP band from ACD‐Me exhibited relatively stronger intensity compared to the acceptor emission band in photonic crystals containing black MCs.

### 2.4 Thermochromic Switching between Structural and Pigment Colors

**Figure 5:**
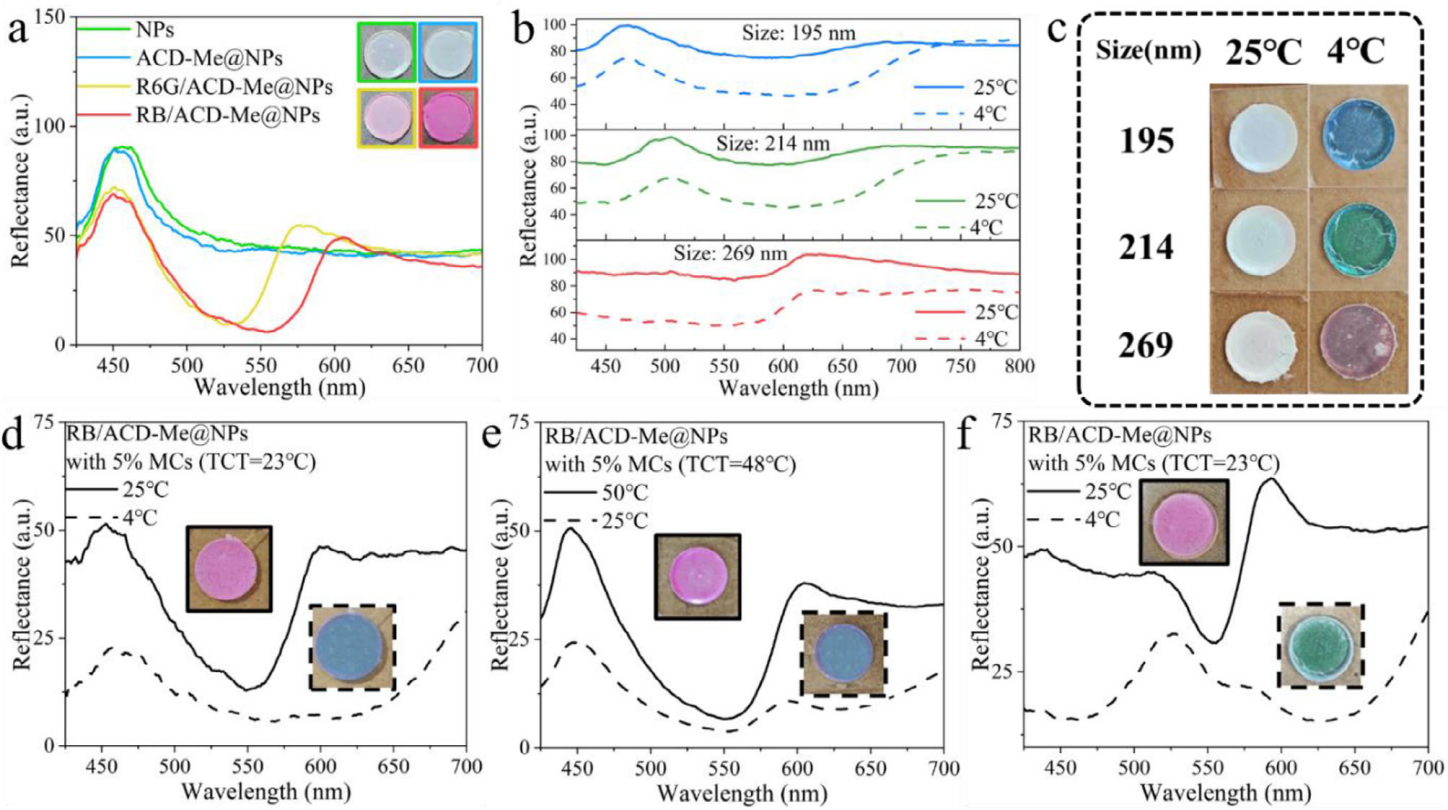
a) Normal‐incident reflection spectra of bare NPs, ACD‐Me@NPs, R6G/ACD‐Me@NPs, and RB/ACD‐Me@NPs, with corresponding digital images shown in the upper right corner. b) Reflectance spectra of NPs with diameters of 195, 214, and 269 nm (with 5% MCs) at 4 and 25 °C. c) Digital images of NPs with diameters of 195 nm, 214 nm, and 269 nm (with 5% MCs) at both temperatures. d) Reflectance spectra of RB/ACD‐Me@NPs (with 5% MCs, TCT = 23 °C) at 4 °C and 25 °C, showing blue structural color and pink pigment color (digital image displayed above corresponding curve). e) Reflectance spectra of RB/ACD‐Me@NPs (with 5% MCs, TCT = 48 °C) at both temperatures, exhibiting blue structural color and pink pigment color (digital image displayed above corresponding curve). f) Reflectance spectra of RB/ACD‐Me@NPs (with 5% MCs, TCT = 23 °C) at both temperatures, displaying green structural color and pink pigment color (digital image displayed above corresponding curve).

Moreover, the MCs induced a thermochromic revelation of the structural color. Despite the strong photonic stop band detected on the surface of the assembled NPs (Figures S17 and S18, Supporting Information), the sample appeared white due to significant incoherent scattering under diffuse reflection observation conditions, which obscured the structural color (**Figure** **5**a).^[^ ^12^ ^]^ Upon cooling to temperatures lower than the solid solvent's melting threshold, the MCs exhibited a darkening transition, concurrently suppressing the problematic light‐scattering phenomenon characteristic of the photonic crystal system, thereby revealing the structural colors (Figure 5b,c). Interestingly, in contrast to the white appearance of assembled NPs governed by incoherent scattering, the pigment colors of R6G and RB predominantly dictated the optical characteristics of the assembled R6G/ACD‐Me@NPs and RB/ACD‐Me@NPs systems (Figure 5d; Figure S19, Supporting Information). However, below the TCT of the MCs, the black MCs suppressed the incoherent scattering and unveiled the structural colors (Figure 5e,f), albeit with reduced saturation due to residual incorporation of pigment components. The angle‐dependent structural colors at 277 K when compared with the angle‐independent pigment color at 298 K further demonstrates such shift between structural color and pigment color (Figure S20, Supporting Information). As shown in the reflectance spectra in Figure 5d,f (FL band included), the reflectance dip (corresponding to the absorption band of RB) was much stronger than the structural resonance band of the assembled RB/ACD‐Me@NPs at temperatures above the TCT of the MCs, whereas the structural resonance band dominated the reflectance spectra at temperatures below the TCT. This thermochromic shift between the structural resonance and pigment colors, utilizing identical R6G and RB doping concentrations for the afterglow energy transfer process (Figure S21, Supporting Information), enabled effective tri‐state thermo‐responsive color modulation. In summary, based on distinct TCTs of MCs, systems can be engineered to exhibit color changes triggered either by heating from room temperature or cooling from room temperature (Figure 5d,e). Simultaneously, pigment‐based coloration and structural coloration can be independently regulated through molecular design of dyes and precise control of NP sizes (Figure 5e and f), respectively, achieving highly controllable thermochromic properties.

### 2.5 Dynamic Anti‐Counterfeiting with Tri‐State Thermo‐Programmable Afterglow Patterns

**Figure 6:**
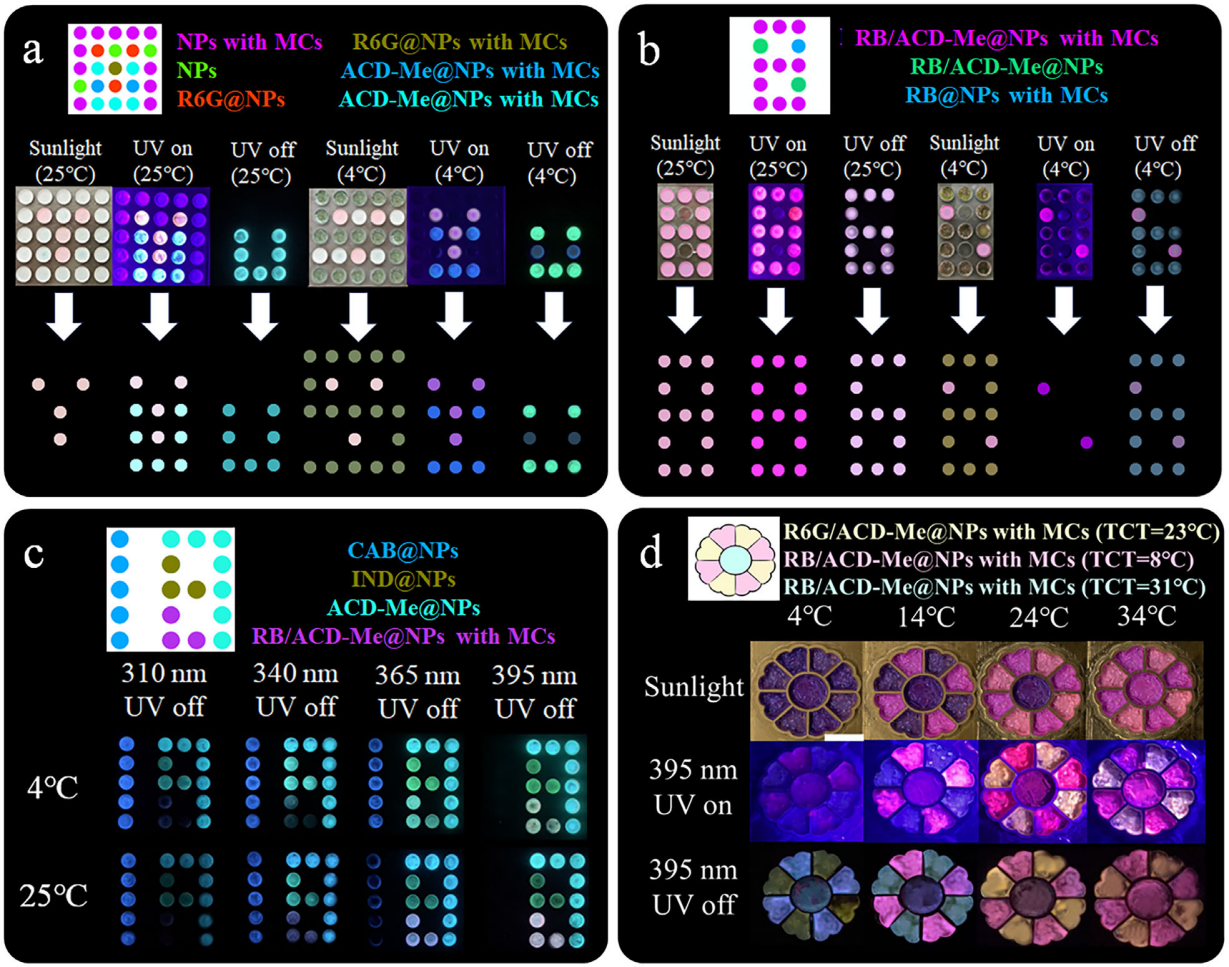
a) Schematic diagram of an alphabetic information encryption matrix with corresponding digital images under: sunlight (4 and 25 °C), UV excitation (λ_ex_ = 395 nm; 4 and 25 °C), and after UV turn‐off (4 and 25 °C). b) Schematic diagram of a numerical information encryption matrix with corresponding digital images under the same illumination and temperature conditions. c) Schematic diagram and digital images of an encryption matrix incorporating different phosphorescent molecules at 4 and 25 °C after UV turn‐off, showing responses to multiple excitation wavelengths (λ_ex_ = 310 nm, 340 nm, 365 nm, and 395 nm). d) Schematic diagram of flower‐patterned matrices with different TCTs, demonstrating color changes under sunlight, UV excitation (λ_ex_ = 395 nm), and after UV turn‐off at various temperatures (4, 14, 24, and 34 °C). Scale bar: 1 cm. MCs weight fraction: 5% relative to the NPs.

Using the full color FL and afterglow of the doped NPs system (Figure S22, Supporting Information), photonically‐engineered security features were fabricated based on the thermo‐responsive tri‐state emission characteristics (**Figure** **6**). As demonstrated in Figure 6a,b, these arrays exhibit pronounced thermochromic structural coloration combined with FL and phosphorescence, generating six distinct optical patterns through temperature modulation (4–34 °C) and light source variation. Notably, utilizing the different excitation ranges of ACD‐Me@NPs, CAB@NPs, and IND@NPs (Figure 6c), the unique stimulus‐responsive nature of the system was further exploited for advanced encryption applications. Specifically, the afterglow chromaticity demonstrated dual responsiveness to both excitation wavelength and temperature. It should be noticed that IND@NPs display both temperature‐ and excitation‐wavelength‐dependent afterglow emission (Figures S23–S26, Supporting Information). The temperature dependence of IND@NPs originates from DF emission, as shown in Figure S6 (Supporting Information), whereas the excitation‐wavelength‐dependent afterglow of IND@NPs likely stems from their dual emission characteristics (dispersed and aggregated states). This enabled the creation of multi‐layered security patterns through strategic doping with diverse phosphors (Figure 6c). Furthermore, MCs with tailored TCTs endowed the system further incremental afterglow color progression (Figure 6d). As illustrated in Figure 6d, FL and structural color activation proceed stepwise, whereas phosphorescence exhibits chromatic shifts across the tested temperature range due to hierarchically activated microcapsules. This mechanism establishes orthogonal information dimensions for cryptographic systems.

## 3 Conclusion

We have demonstrated a tri‐state photonic crystal film by incorporating organic persistent luminescent molecules into a PMMA‐based 3D photonic crystal architecture through solid‐phase extraction. The resulting advanced anti‐counterfeiting material demonstrated three distinct optical states: fluorescence, phosphorescence, and structural coloration, effectively eliminating counterfeit possibilities. Furthermore, we achieved thermochromic regulation across all three optical states through thermo‐responsive MCs that modulate both far‐field light absorption and near‐field TS‐FRET. In typical TS‐FRET, higher energy transfer efficiency shortens the afterglow lifetime. In contrast, far‐field light absorption avoids this trade‐off, preserving the long‐lived triplet emission. By using such far‐field light absorption characteristics, hierarchical security labeling systems with embedded data encryption can be further engineered through integration with other photonic crystal architectures and thermally responsive technologies.^[^ ^13^ ^]^ The unique optical modulation capabilities enable precise engineering of photonic structures with tunable light‐matter interactions, exhibiting dynamic visual responses under specific illumination conditions. These programmable optical characteristics establish the technology as a high‐security authentication feature for product protection applications.

## 4 Experimental Section

### Synthesis of NPs

Nanoparticle synthesis was performed with modifications to previously reported soap‐free emulsion polymerization methods.^[^ ^14^ ^]^ A mixture containing 10.8 mL methyl methacrylate, 1.2 mL α‐methacrylate, and 80 mL deionized water was gradually added to a 100 mL round‐bottom flask, followed by the addition of 500–2000 µL 1 wt.% sodium dodecyl sulfate (SDS; nanoparticle size was controlled by SDS dosage) solution. The flask was immersed in a water bath and heated to 78 °C. Separately, 0.1 g ammonium persulfate was dissolved in 2 mL deionized water to serve as the initiator. Upon temperature stabilization at 78 °C, the initiator solution was added dropwise to the reaction mixture under continuous stirring. After 8 h reaction, the crude product was centrifuged at 11,000 rpm for 60 min. The supernatant was decanted, and the precipitate was redispersed in deionized water via sonication. This centrifugation‐redispersion cycle was repeated three times, followed by adjustment to 10% solid content for subsequent use.

### Synthesis of ACD‐Me@NPs

ACD‐Me‐loaded nanoparticles were prepared using a solid‐phase extraction method. 20 g purified NP suspension was mixed with 0.1 g ACD‐Me and oscillated for 12 h. After removing unencapsulated ACD‐Me through initial centrifugation (5,000 rpm, 10 min), the ACD‐Me@NPs‐containing supernatant was further isolated by high‐speed centrifugation (11,000 rpm, 60 min). After removing residual aqueous ACD‐Me in the supernatant, the pellet was redispersed in deionized water through sonication. Three purification cycles were performed before adjusting to 10% solid content (CAD@NPs and IND@NPs were similarly prepared).

### Synthesis of R6G/ACD‐Me@NPs and RB/ACD‐Me@NPs

R6G/ACD‐Me@NPs and RB/ACD‐Me@NPs were fabricated via an ion‐exchange process. R6G or RB was dissolved in deionized water (1 mg mL^−1^) and incrementally added to ACD‐Me@NPs suspension to achieve desired doping ratios. The final product was concentrated to 10% solid content through centrifugation‐redispersion cycles.

### Fabrication of Thermoresponsive Microcapsules

The thermosensitive microcapsules was prepared using an emulsion templating approach with gelatin as the stabilizer.^[^ ^11^ ^]^ Briefly, solid solvents (1‐detanol for TCT = 8 °C, 1‐dodecanol for TCT = 23 °C, 1‐tridecanol for TCT = 31 °C, 1‐hexadecanol for TCT = 48 °C; 2 g), bisphenol A (0.1 g), ODB‐2 (0.1 g), and PMMA (0.6 g) were combined in 15 mL of dichloromethane to form a homogeneous organic phase. This precursor solution was subsequently emulsified through dropwise addition into an aqueous gelatin solution (2 wt.%) while maintaining high shear mixing (8000 rpm) for 20 min at ambient conditions. Following initial emulsification, the system was maintained at 35 °C with moderate agitation (400 rpm) for 12 h to allow complete solvent removal. The solidified microcapsules were then isolated via centrifugal separation, subjected to multiple deionized water rinses, and vacuum‐dried to yield the final particulate material.

### Synthesis of the Photonic Crystal Array of NPs

NPs suspension with MCs was cast into mold cavities and air‐dried. A 5% ascorbic acid aqueous solution (oxygen‐consuming agent to enable afterglow emission) was spray‐coated onto the matrix. The assembly was vacuum‐sealed and aged for 6 h to complete pattern formation (ACD‐Me@NPs, CAD@NPs, IND@NPs, R6G/ACD‐Me@NPs, and RB/ACD‐Me@NPs matrices were prepared identically).

## Conflict of Interest

The authors declare no conflict of interest.

## Supporting information



Supporting Information

## Data Availability

The data that support the findings of this study are available from the corresponding author upon reasonable request.
