# Supplementary material for: Thermo‐Responsive Tri‐State Photonic Crystals
Source: Adv Sci (Weinh). 2025 Jun 4;12(32):e06507. doi: 10.1002/advs.202506507 (PMC12407271; doi:10.1002/advs.202506507)
Supplement: Supplementary file 1 — Supporting Information [file ADVS-12-e06507-s001.pdf]

ADVANCED SCIENCE
Open Access

Supporting Information

for *Adv. Sci.*, DOI 10.1002/advs.202506507

Thermo-Responsive Tri-State Photonic Crystals

*Yuewei Zheng, Jinqing Chen, Wanqi Mo and Wei Hong**

Supporting Information

Thermo-Responsive Tri-State Photonic Crystals

*Yuewei Zheng, Jinqing Chen, Wanqi Mo, Wei Hong**

Key Laboratory for Polymeric Composite and Functional Materials of Ministry of Education, School of Chemistry, Sun Yat-sen University, Guangzhou 510275, P. R. China

E-mail: hongwei9@mail.sysu.edu.cn

Table of Content

1. Materials and Characterization 1
1.1 Materials 1
1.2 Characterization 1
1.3 Theoretical Calculation of Average Scattering Free Path 2
1.3.1 Time-dependent density functional theory (TD-DFT) simulation for ACD-Me 2
1.3.2 Pure NPs photonic crystal.. 2
1.3.3 NPs photonic crystals containing microcapsules (MCs) 3
1.3.4 Calculation of refractive index of NPs at different incident wavelengths 4
2. Results and Discussion.. 6
2.1Supplementary figures 6
2.2 Supplementary tables 27
2.3 Supplementary MATLAB code 28
2.3.1 Theoretical calculation code for the average scattering free path of pure NPs photonic crystals 28
2.3.2 Theoretical calculation code for the average scattering free path of NPs photonic crystals with microcapsules.. 30
3. References 32

1. Materials and Characterization

1.1 Materials

Methylacrylic acid (MAA) and ammonium persulfate (>99.0%, electrophoretic grade) were purchased from Macklin Biochemical Co., Ltd (Shanghai, China). N-Methyl-9-acridone (>98%) and styrene (>99.0%, stabilized with TBC) were purchased from TCI Chemical Trading Co., Ltd (Shanghai, China). Ascorbic acid and methyl methacrylate (>99.0%) were purchased from Aladdin Biochemical Technology Co., Ltd (Shanghai, China). 9-(2-Carboxyphenyl)-3,6-bis(diethylamino)xanthylium chloride (RB), 9-(2-(ethoxycarbonyl) phenyl)-3,6-bis(ethylamino)-2,7-dimethylxanthylium chloride (R6G), 11,12-dihydroindolo[2,3-a] carbazole and 9H-pyrido[3,4-b] indole was purchased from Bide Pharmaceutical Technology Co., Ltd (Shanghai, China).

1.2 Characterization

SEM images were obtained on a field emission scanning electron microscopy (FE-SEM, Hitachi S-4800). The reflectance spectra were measured on a spectrophotometer (DH-2000-BAL, Ocean Optics) equipped with a reflection probe. Steady-state spectra, delayed emission spectra, and lifetimes were measured using an Edinburgh Instruments LTD FLS980 spectrofluorometer. PL quantum yields (PLQYs) were recorded using a Hamamatsu C9920 PLQY measurement system equipped with an integrating sphere (φ=150 mm). X-ray diffraction (XRD) patterns were collected using a Rigaku Co SmartLab with CuKα radiation. The UV absorption spectrum was measured by a UV visible near-infrared spectrophotometer (Lambda 950, Perkin Elmer).

1.3 Theoretical Calculation of Average Scattering Free Path [1]

1.3.1 Time-dependent density functional theory (TD-DFT) simulation for ACD-Me

The TD-DFT calculation was performed on Gaussian 16 program.[2] The ground states of the molecules were optimized by DFT calculation with B3LYP/def2tzvp basis set. The excited states investigation was calculated by TD-DFT calculation with B3LYP/def2tzvp basis set. Spin-orbit coupling matrix elements (SOCME) were evaluated with PySOC,[3] which called the MolSOC code to calculate the atomic integrals. Parameters for the effective charge in the operator for the atomic integrals were taken from the MolSOC code,[4] without further optimization.

1.3.2 Photonic crystals formed by NPs

The average scattering free path is closely related to the structure of the photonic crystal, the photon wavelength, and the scattering mechanism. The mean free path (MFP) of a photonic crystal depends on both its structural characteristics and the scattering behavior of photons. The MFP (ℓ_s) can be calculated using the following formula:[5]

$$\ell_s = \frac{1}{\rho \cdot \sigma} \tag{S1}$$

Here, **ρ** is the density of scattering centers in the photonic crystal (i.e., the number of scattering centers per unit volume), which depends on structural defects, disorder, or impurities. In an ideal photonic crystal, **ρ** is typically lower, whereas in a disordered photonic crystal, **ρ** is higher. The value of **ρ** can be calculated as:[5]

$$\rho = \frac{N}{V} \tag{S2}$$

where **N** is the total number of scattering centers, and **V** is the volume of the photonic crystal.

σ is the scattering cross-section of a photon, which depends on the photon wavelength, the dielectric constant distribution of the photonic crystal, and the scattering mechanism (e.g., Rayleigh scattering, Mie scattering). It can be

determined through theoretical models or experimental measurements. Mie scattering theory is particularly suitable for spherical particles and can be used to calculate the scattering cross-section (**σ**) of scatterers in photonic crystals:[6]

$$\sigma = Q_{sca} \cdot \pi a^2 \tag{S3}$$

Here, $\mathbf{Q_{sca}}$ is the scattering efficiency, and **a** is the radius of the scatterer (e.g., spherical particles). The $\mathbf{Q_{sca}}$ can be calculated using the Mie scattering coefficients $\mathbf{a_n}$ and $\mathbf{b_n}$:[7]

$$Q_{sca} = \frac{2}{x^2}\sum_{n=1}^{\infty}(2n+1)(|a_n|^2 + |b_n|^2) \tag{S4}$$

where **x** is the size parameter, given by:[8]

$$x = \frac{2\pi a n_m}{\lambda} \tag{S5}$$

Here, **λ** is the wavelength of the incident wave, and $\mathbf{n_m}$ is the refractive index of the background medium.

The Mie scattering coefficients $\mathbf{a_n}$ and $\mathbf{b_n}$ are calculated as follows:

$$a_n = \frac{m\psi_n(mx)\psi_n'(x) - \psi_n(x)\psi_n'(mx)}{m\psi_n(mx)\xi_n'(x) - \xi_n(x)\psi_n'(mx)} \tag{S6}$$

$$b_n = \frac{\psi_n(mx)\psi_n'(x) - m\psi_n(x)\psi_n'(mx)}{\psi_n(mx)\xi_n'(x) - m\xi_n(x)\psi_n'(mx)} \tag{S7}$$

where $\boldsymbol{\psi_n}$ and $\boldsymbol{\xi_n}$ are Riccati-Bessel functions, and $\boldsymbol{\psi_n}'$ and $\boldsymbol{\xi_n}'$ are their derivatives.

1.3.3 NPs photonic crystals containing microcapsules (MCs)

When microcapsules are introduced, the photonic crystal system no longer consists solely of NP microspheres, and the relative content of microspheres and microcapsules must be considered. Their relative proportions can be quantified by their volume fractions. Due to differences in density and scattering cross-sections between microcapsules and NP microspheres, the average scattering free path (ℓ_s) is adjusted as follows:

$$\ell_s = \frac{1}{\rho \cdot \langle\sigma\rangle} \tag{S8}$$

Here, **ρ** is the total scattering center density:[9]

$$\rho = \rho_1 + \rho_2 \tag{S9}$$

where:

- **ρ_1** is the density of NP microspheres,
- **ρ_2** is the density of microcapsules.

These are calculated as:

$$\rho_1 = \frac{f_1}{\frac{4}{3}\pi a_1^3} \tag{S10}$$

$$\rho_2 = \frac{f_2}{\frac{4}{3}\pi a_2^3} \tag{S11}$$

Here, **f_1** and **f_2** are the volume fractions of NP microspheres and microcapsules, respectively, while **a_1** and **a_2** are their respective radii.

$\langle\sigma\rangle$ is the weighted average of the total scattering cross-section, given by:[10]

$$\langle\sigma\rangle = \frac{\rho_1\sigma_1+\rho_2\sigma_2}{\rho_1+\rho_2} \tag{S12}$$

where:

- **σ_1** is the scattering cross-section of NP microspheres,
- **σ_2** is the scattering cross-section of microcapsules.

These are calculated as:

$$\sigma_1 = Q_{sca,\ 1} \cdot \pi a_1^2 \tag{S13}$$

$$\sigma_2 = Q_{sca,\ 2} \cdot \pi a_2^2 \tag{S14}$$

The methods for calculating **$Q_{sca,1}$** and **$Q_{sca,2}$** are the same as described earlier.

1.3.4 Calculation of refractive index of NPs at different incident wavelengths

The refractive index of NPs exhibits dispersion phenomenon at different incident light wavelengths, that is, the refractive index gradually decreases with increasing wavelength. This change follows the typical dispersion law of transparent materials and can be described by the empirical formula Cauchy equation. The following is a specific analysis:

$$n = A + \frac{B}{\lambda^2} + \frac{C}{\lambda^4} \tag{S15}$$

For NPs (similar to pure PMMA):

- **A**≈1.488
- **B**≈3.00×10^3 nm^2
- **C**≈-2.50×10^8 nm^4

The calculation results are shown in Tables S2 and S3.

2. Results and Discussion

2.1 Supplementary figures

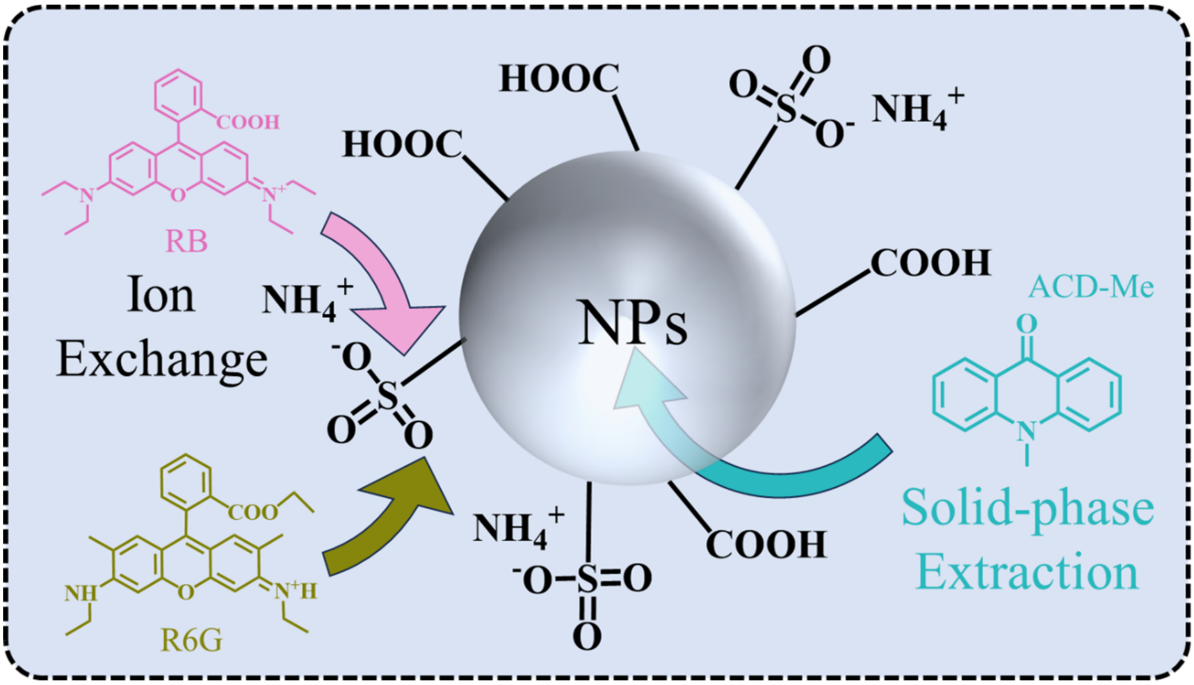

Scheme S1. Schematic diagram of ACD-Me and R6G/RB absorbed into NPs.

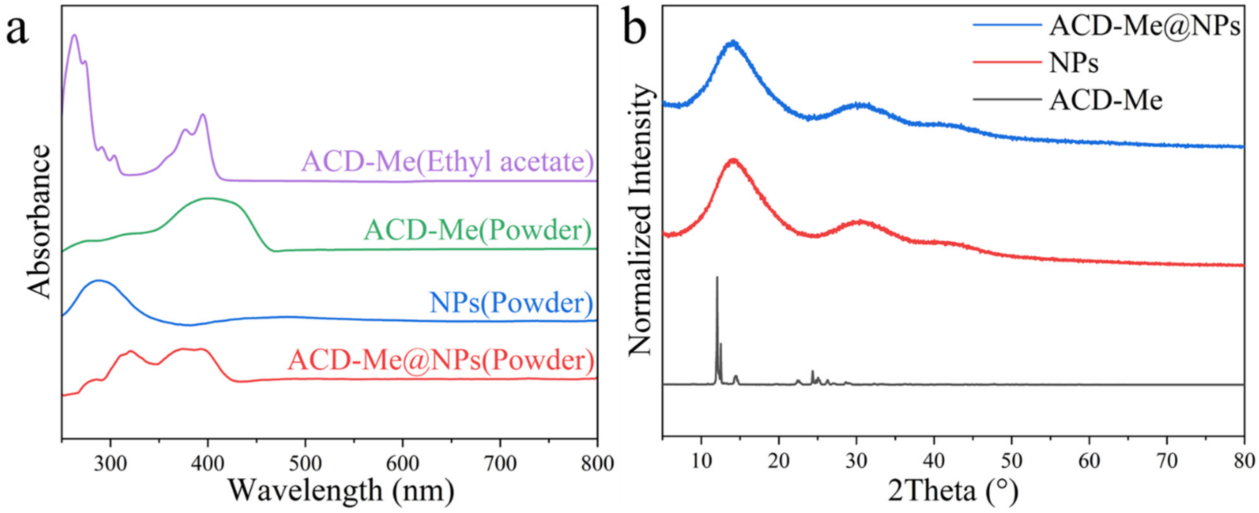

Figure S1. (a) UV absorption spectra of ACD-Me (dissolved in ethyl acetate), ACD-Me (powder), NPs (powder), and ACD-Me@NPs (powder); (b) X-ray diffraction patterns of ACD-Me, NPs, and ACD-Me@NPs.

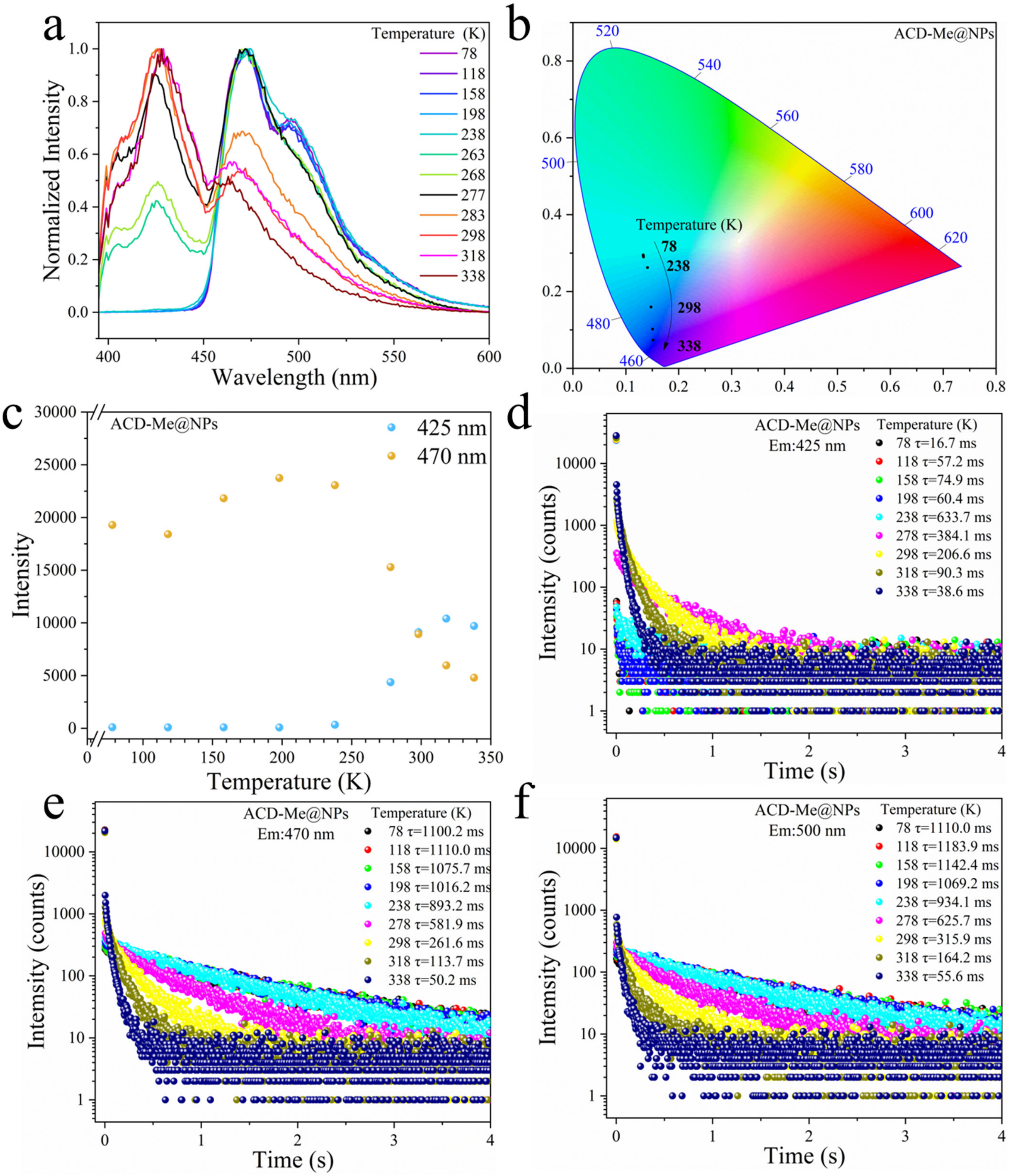

Figure S2. (a) Delayed PL spectra of ACD-Me@NPs at various temperatures; (b) CIE coordinate diagrams of the delayed emission from ACD-Me@NPs at different temperatures; (c) Delayed PL intensities at 425 nm and 470 nm; (d–f) Lifetime decay profiles of ACD-Me@NPs monitored at (d) 425 nm, (e) 470 nm, and (f) 500 nm.

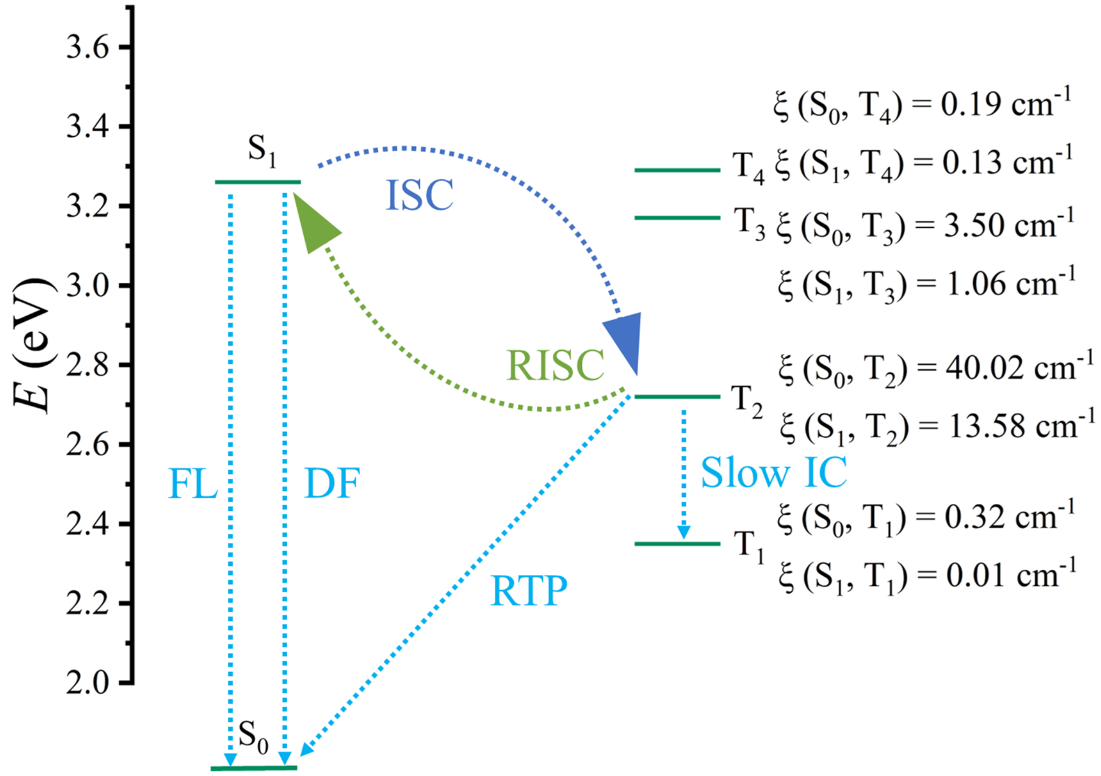

Figure S3. Diagram of the calculated energy levels of ACD-Me.

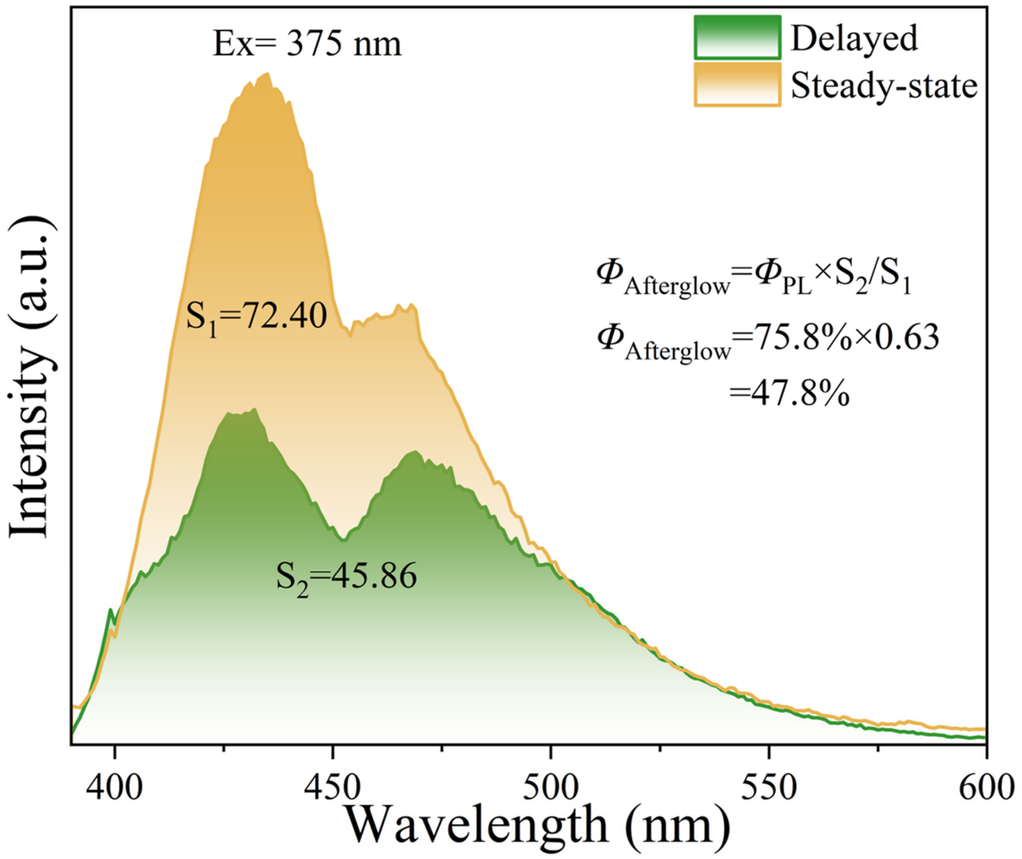

Figure S4. The afterglow quantum yield of ACD-Me@NPs calculated by the area ratio of steady-state and delayed spectrum.

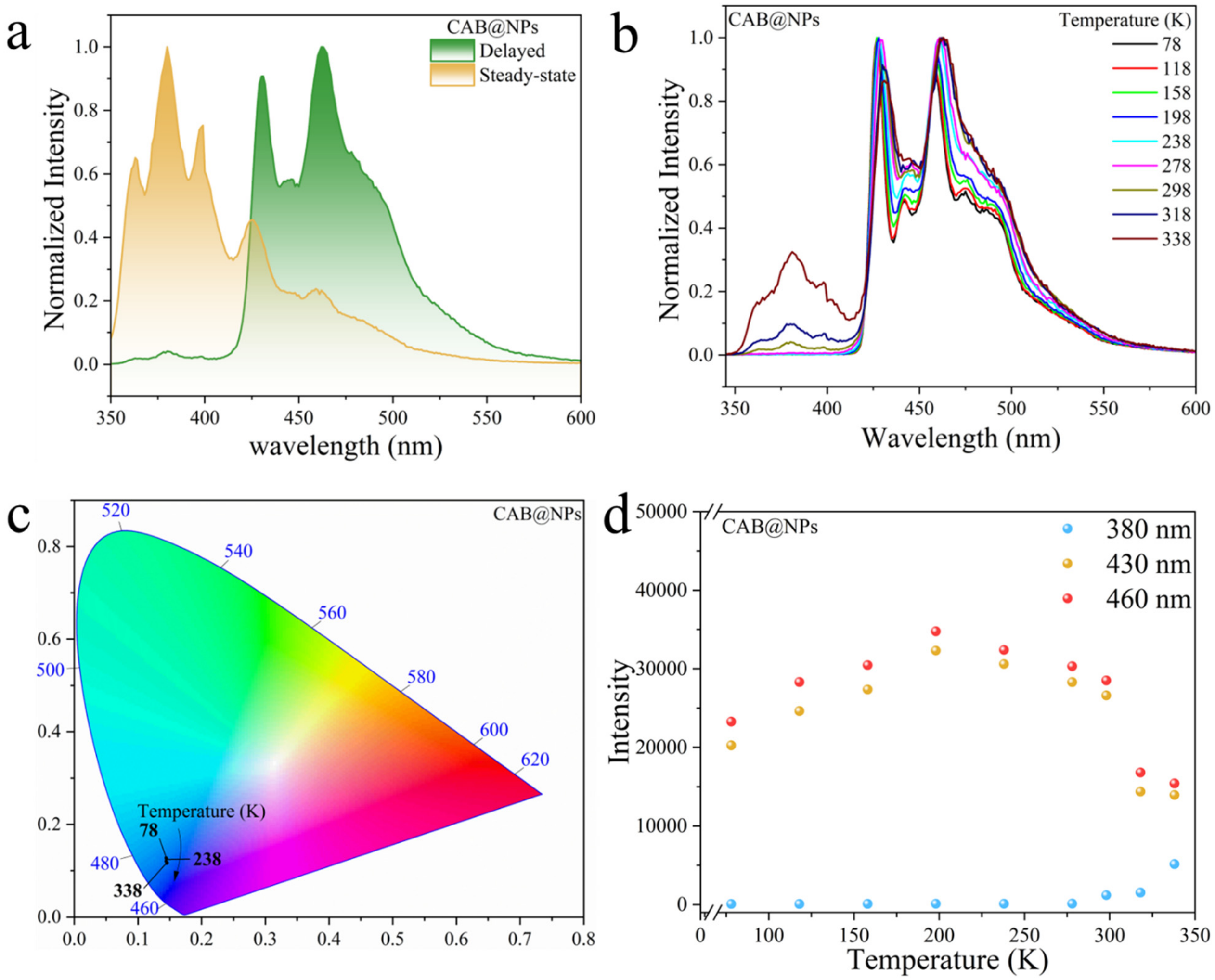

Figure S5. (a) Steady-state and delayed PL spectra of CAB@NPs; (b) Temperature-dependent delayed PL spectra of CAB@NPs; (c) CIE chromaticity diagrams showing the delayed emission color of CAB@NPs at different temperatures; (d) Delayed PL intensities monitored at 380 nm, 430 nm, and 460 nm.

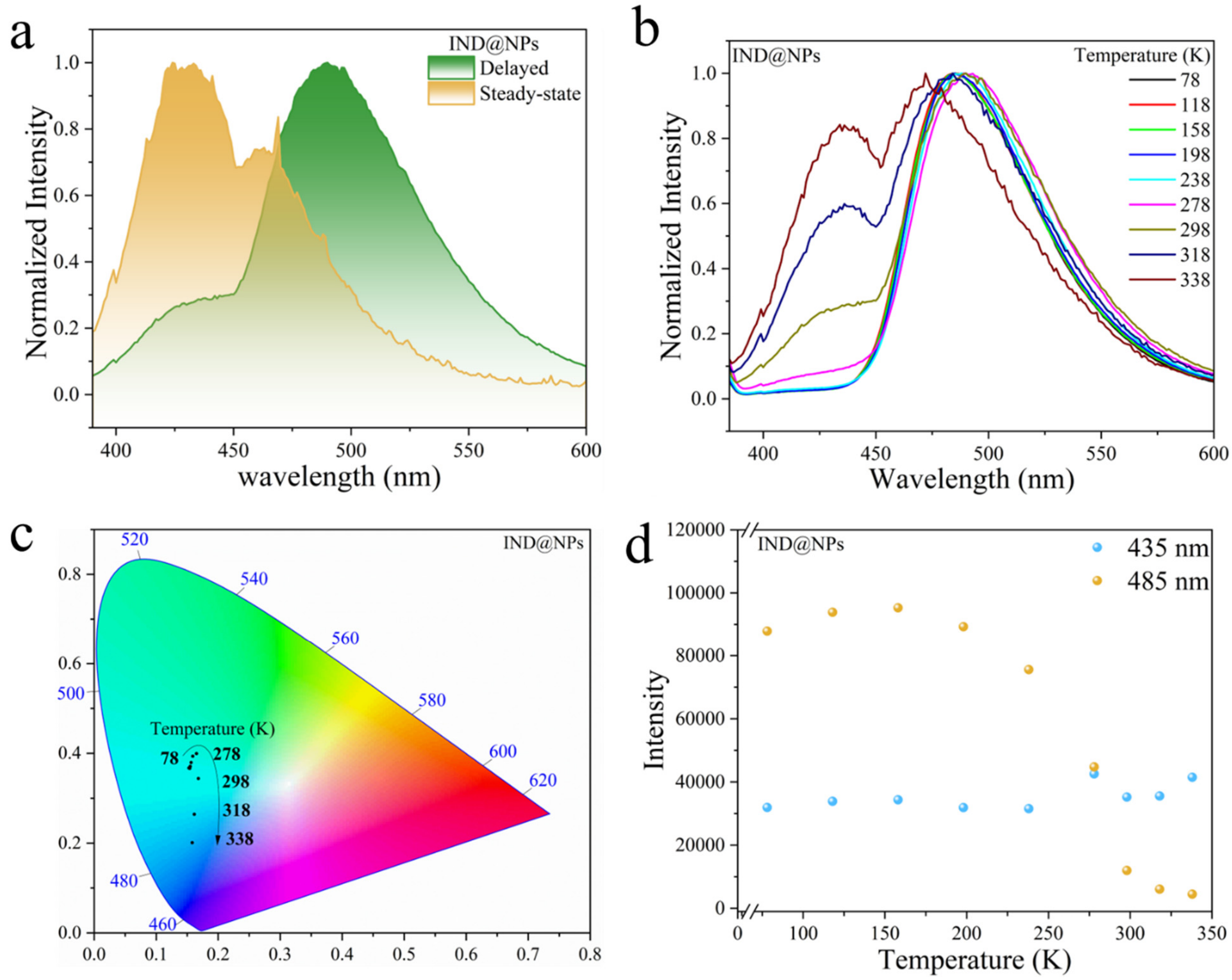

Figure S6. (a) Steady-state and delayed PL spectra of IND@NPs; (b) Temperature-dependent delayed PL spectra of IND@NPs; (c) CIE chromaticity diagrams of IND@NPs at different temperatures; (d) Delayed PL intensities monitored at 435 nm and 485 nm.

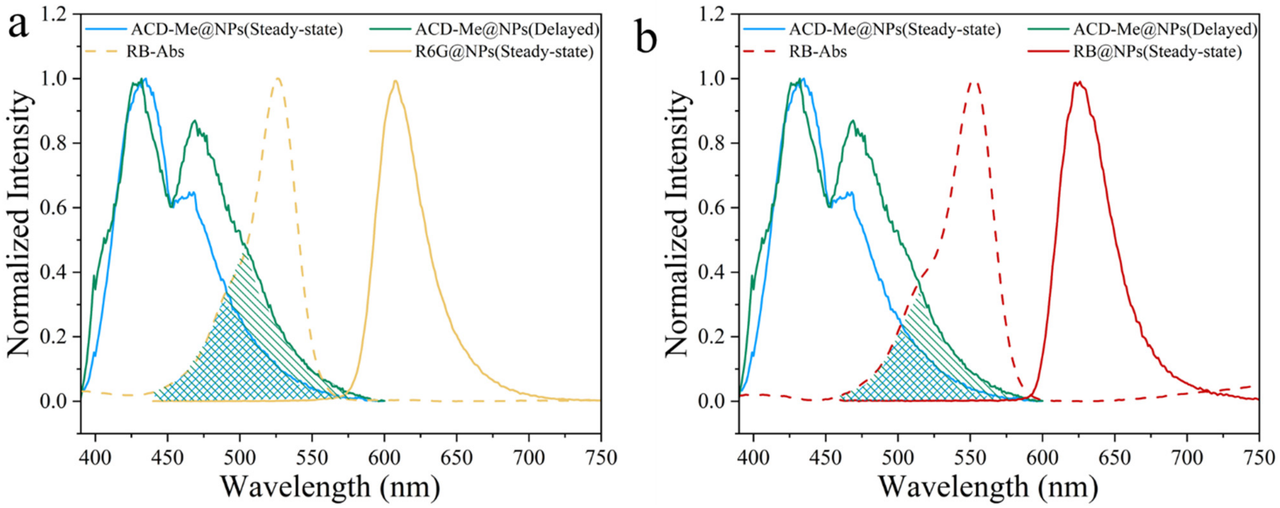

Figure S7. (a) Spectral characterization of ACD-Me@NPs and R6G systems: steady-state PL of ACD-Me@NPs (blue curve), delayed PL of ACD-Me@NPs (green curve), absorbance spectrum of R6G (dashed yellow curve), and PL spectrum of R6G@NPs (yellow curve). Blue-shaded area represents spectral overlap between steady-state PL of ACD-Me@NPs and R6G absorbance; green-shaded area indicates overlap between delayed PL of ACD-Me@NPs and R6G absorbance. (b) Spectral characterization of ACD-Me@NPs and RB systems: steady-state PL of ACD-Me@NPs (blue curve), delayed PL of ACD-Me@NPs (green curve), absorbance spectrum of RB (dashed red curve), and PL spectrum of RB@NPs (red curve). Blue-shaded area shows spectral overlap between steady-state PL of ACD-Me@NPs and RB absorbance; green-shaded area denotes overlap between delayed PL of ACD-Me@NPs and RB absorbance.

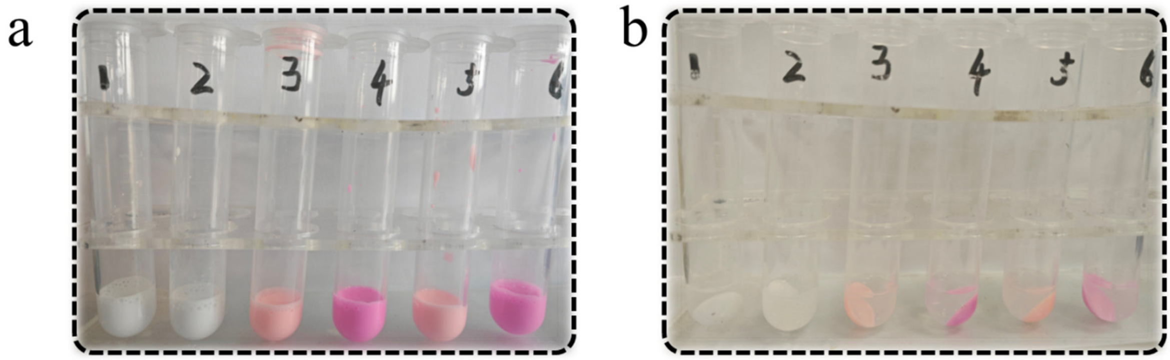

Figure S8. (a) Photographs of six different dispersion before centrifugation; (b) Corresponding samples after centrifugation. Samples 1-6 represent: (1) bare NPs, (2) ACD-Me@NPs, (3) R6G@NPs, (4) RB@NPs, (5) R6G/ACD-Me@NPs, and (6) RB/ACD-Me@NPs, with R6G/RB doping concentrations of 0.075 wt%/0.25 wt%, respectively.

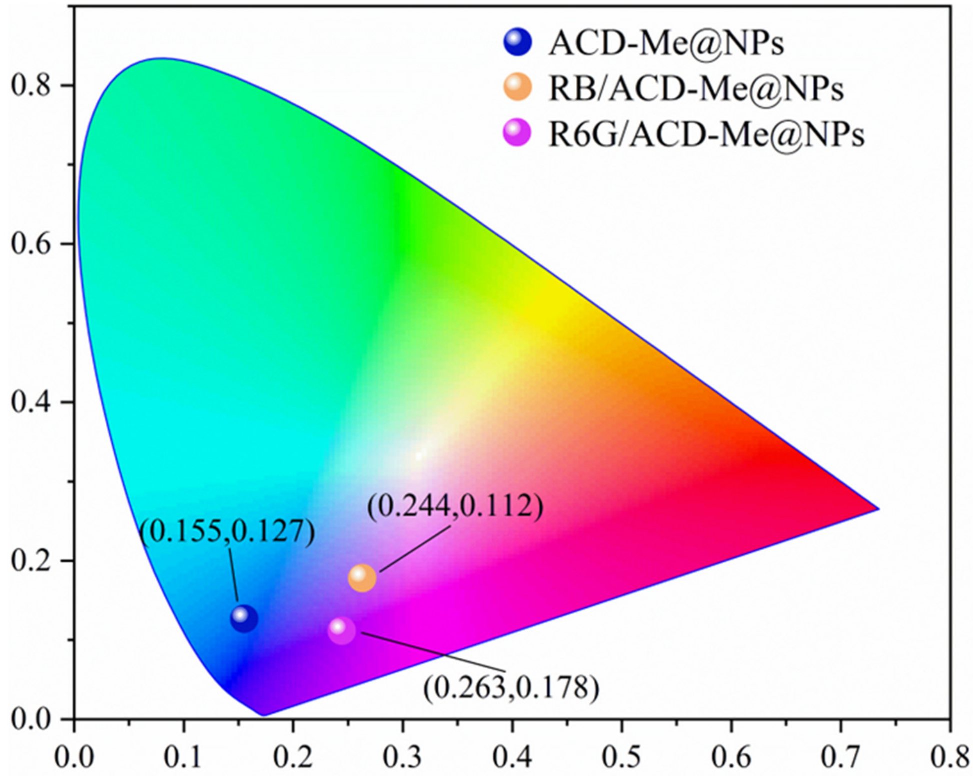

Figure S9. CIE chromaticity diagrams of (1) ACD-Me@NPs, (2) R6G/ACD-Me@NPs, and (3) RB/ACD-Me@NPs, with R6G/RB doping concentrations of 0.075 wt%/0.25 wt%, respectively.

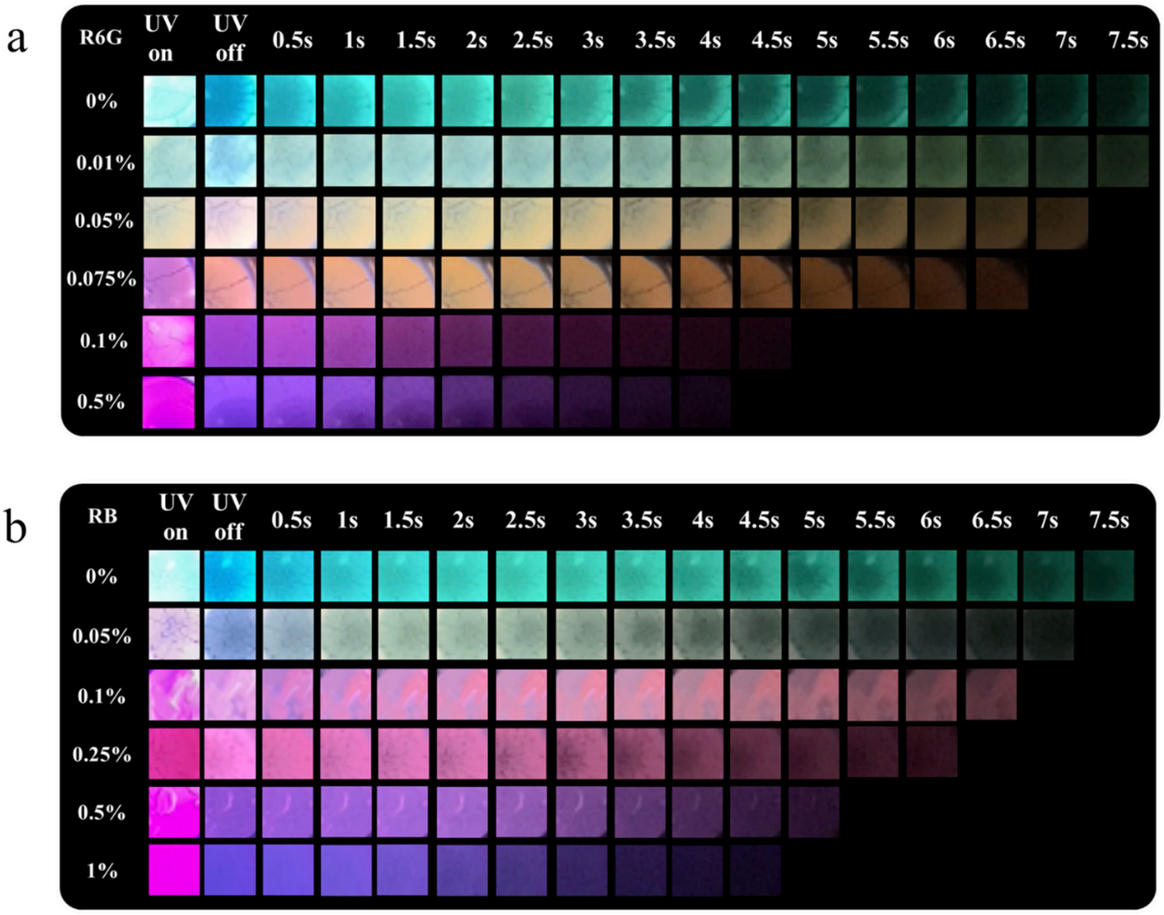

Figure S10. (a) Photographs of R6G/ACD-Me@NPs with varying R6G concentrations by UV excitation (λ_{ex} = 395 nm) on and off; (b) Photographs of RB/ACD-Me@NPs with different RB concentrations by UV excitation (λ_{ex} = 395 nm) on and off.

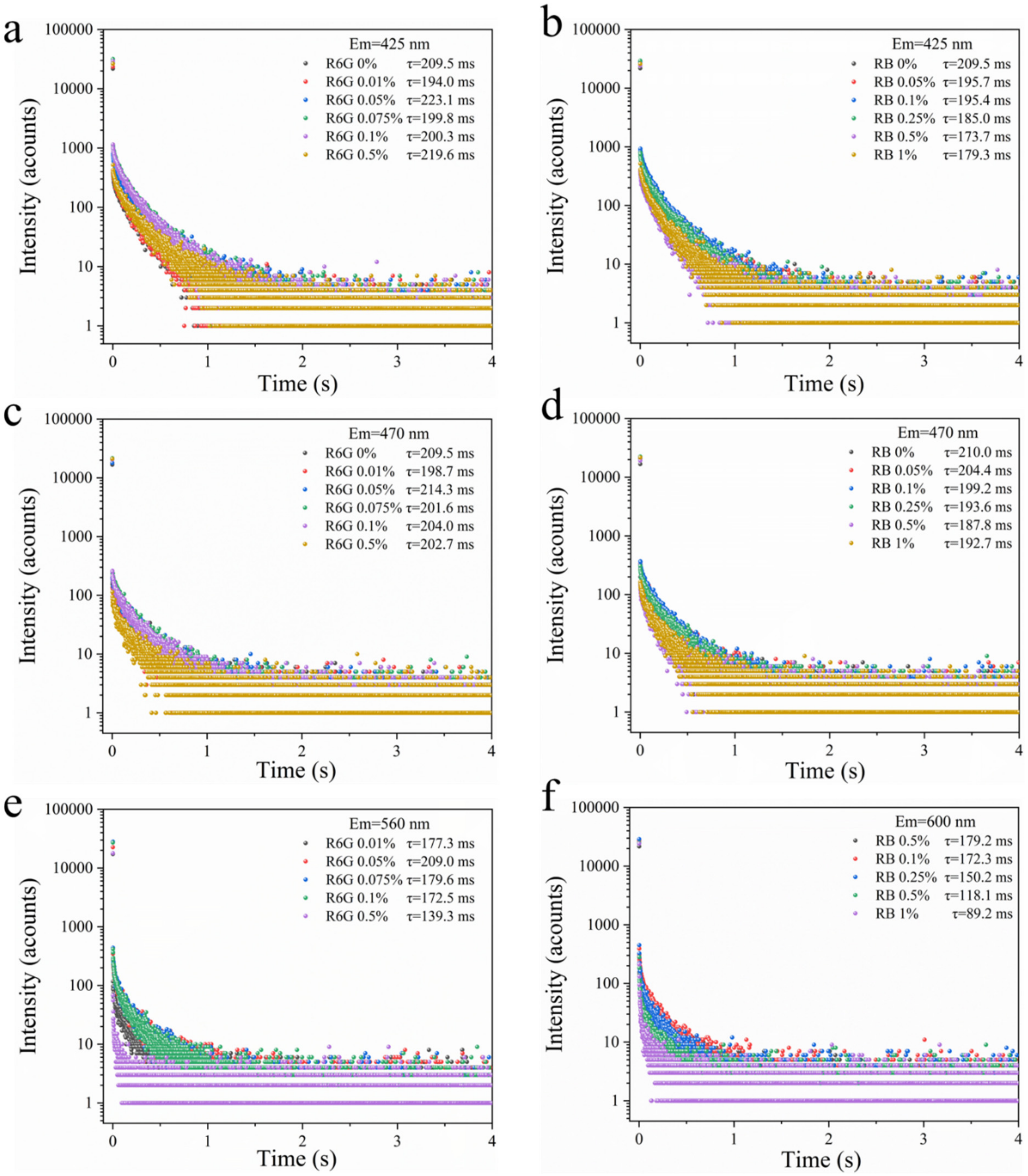

Figure S11. Lifetime decay profiles of (a, c, e) R6G/ACD-Me@NPs monitored at 425 nm, 470 nm, and 560 nm with varying R6G concentrations; (b, d, f) RB/ACD-Me@NPs monitored at 425 nm, 470 nm, and 600 nm with different RB concentrations.

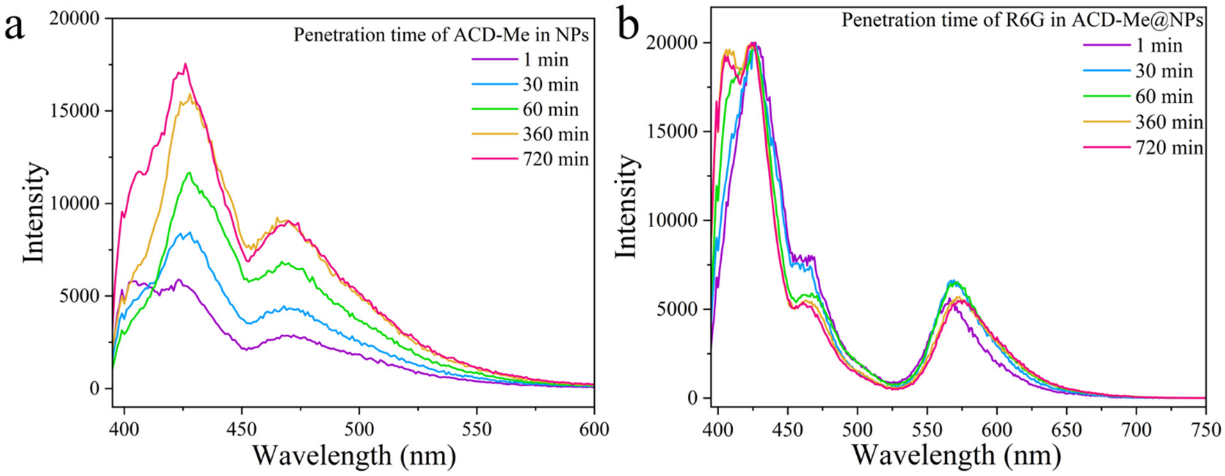

Figure S12. a) Delayed PL spectra of ACD-Me@NPs with different absorption times for ACD-Me; b) Delayed PL spectra of R6G/ACD-Me@NPs with different absorption times for R6G.

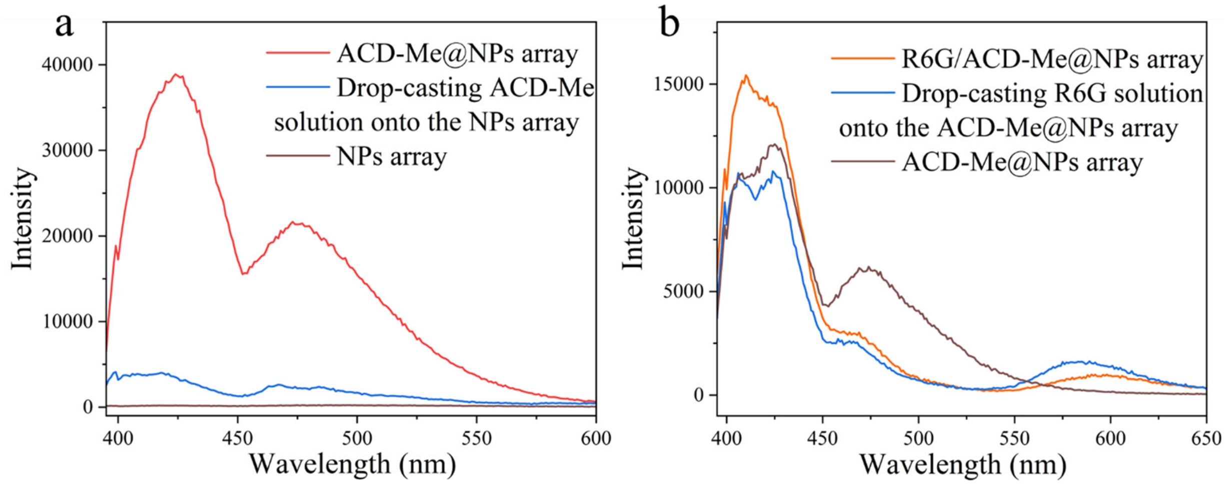

Figure S13. a) Delayed PL spectra of: (i) ACD-Me@NPs, (ii) NPs loaded with ACD-Me by drop-casting (same ACD-Me concentration as (i)), and (iii) pure NPs. b) Delayed PL spectra of: (i) R6G/ACD-Me@NPs, (ii) ACD-Me@NPs loaded with R6G by drop-casting (same R6G concentration as (i)), and (iii) pure ACD-Me@NPs.

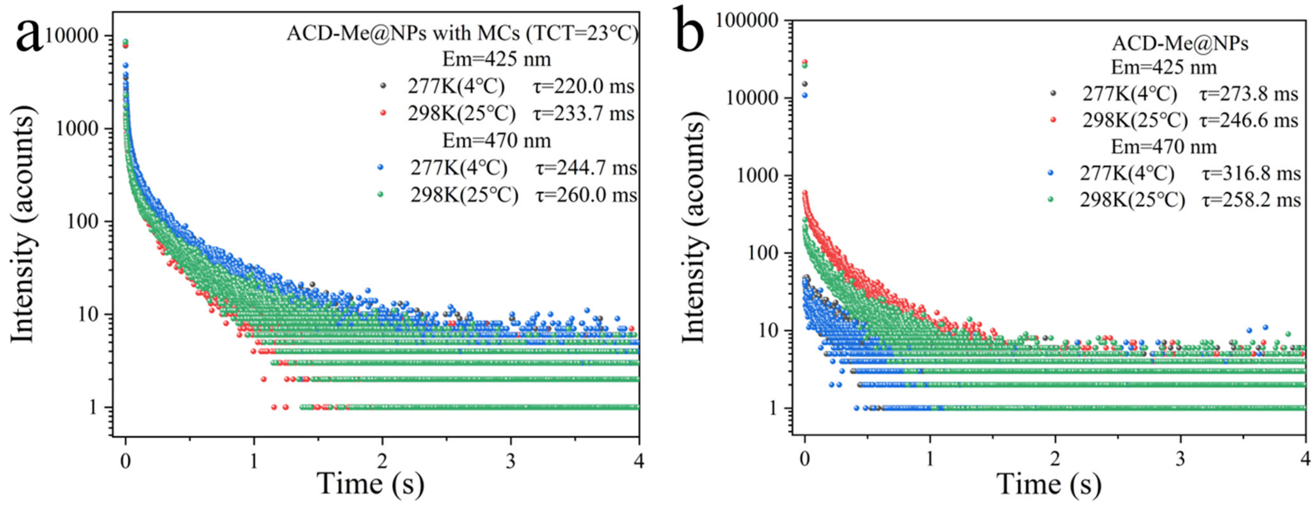

Figure S14. (a) Lifetime decay profiles of ACD-Me@NPs (with 5% MCs, TCT=23°C) monitored at 425 nm and 470 nm under 4 °C and 25 °C conditions; (b) Lifetime decay profiles of pure ACD-Me@NPs measured at the same wavelengths and temperatures.

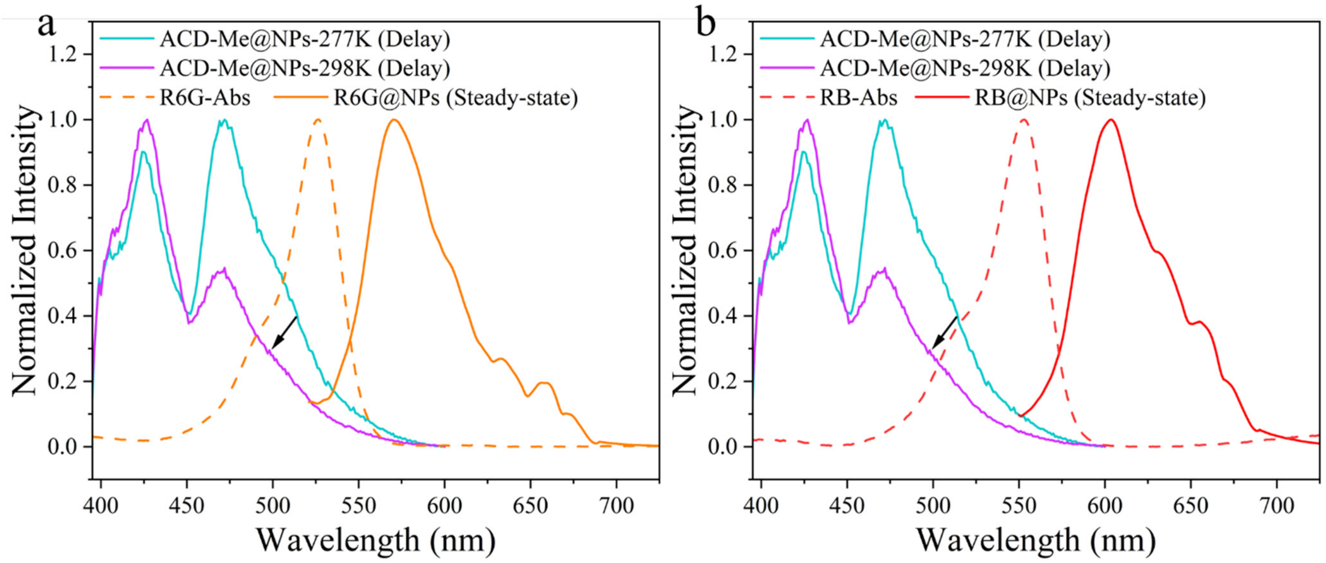

Figure S15 a) Delayed photoluminescence (PL) spectrum of ACD-Me@NPs (bluish-green curve at 277K, purple curve at 298K), absorbance spectra of R6G (dashed orange curve) and PL spectra of R6G@NPs (orange curve); b) Delayed photoluminescence (PL) spectrum of ACD-Me@NPs (bluish-green curve at 277K, purple curve at 298K), absorbance spectra of RB (dashed red curve) and PL spectra of RB@NPs (red curve).

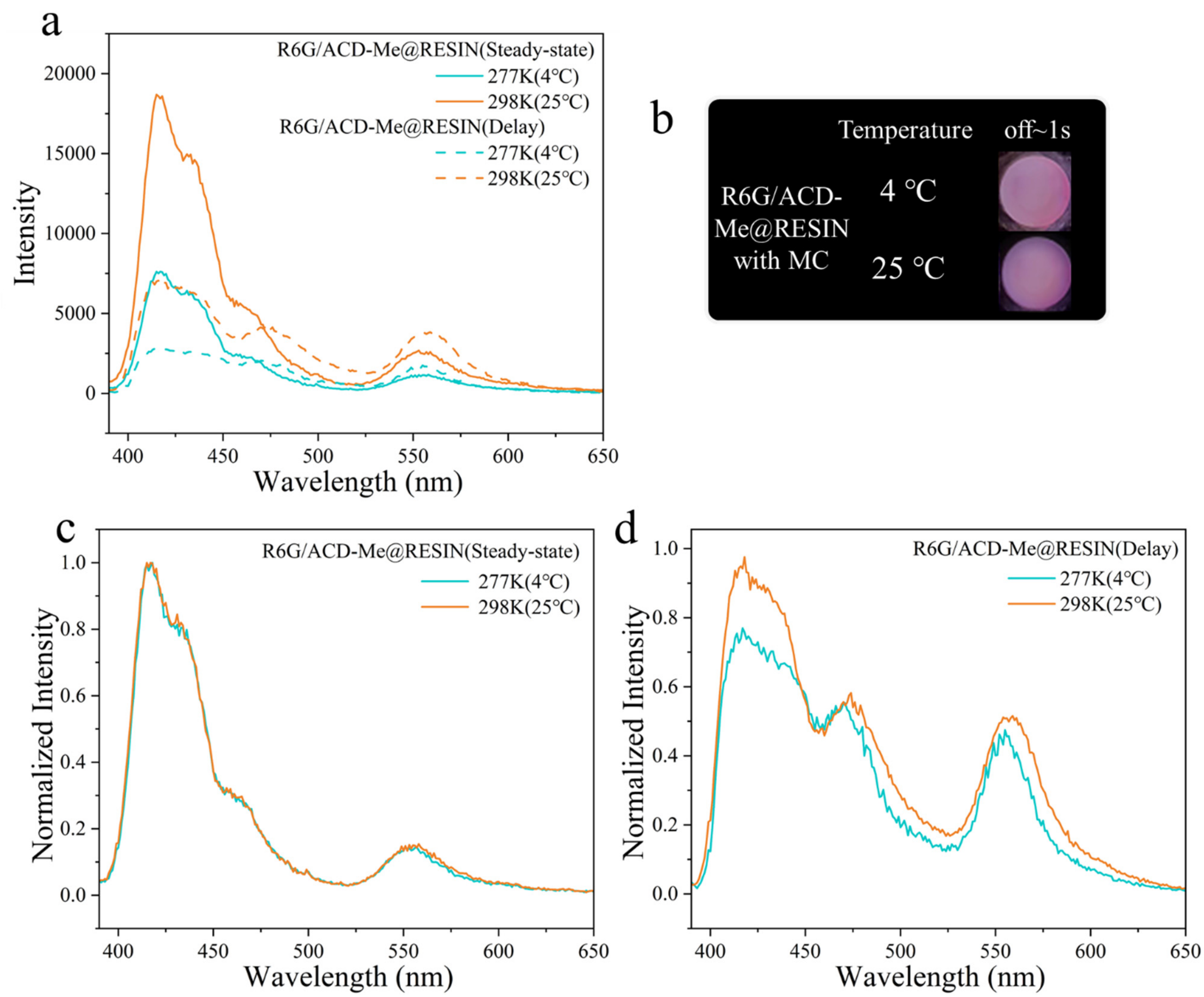

Figure S16. (a) Steady-state and delayed PL spectra of R6G/ACD-Me-doped transparent PMMA resin (R6G/ACD-Me@RESIN) with 5% MCs (TCT=23 °C) under 395 nm excitation at 4 °C and 25 °C (ACD-Me: 0.01 wt% in resin; R6G: 0.075 wt% in ACD-Me@RESIN); (b) Photographs of R6G/ACD-Me@RESIN (5% MCs, TCT=23°C) at both temperatures after ceasing the UV excitation; (c) Temperature-dependent steady-state PL spectra normalized at 470 nm; (d) Temperature-dependent delayed PL spectra normalized at 470 nm.

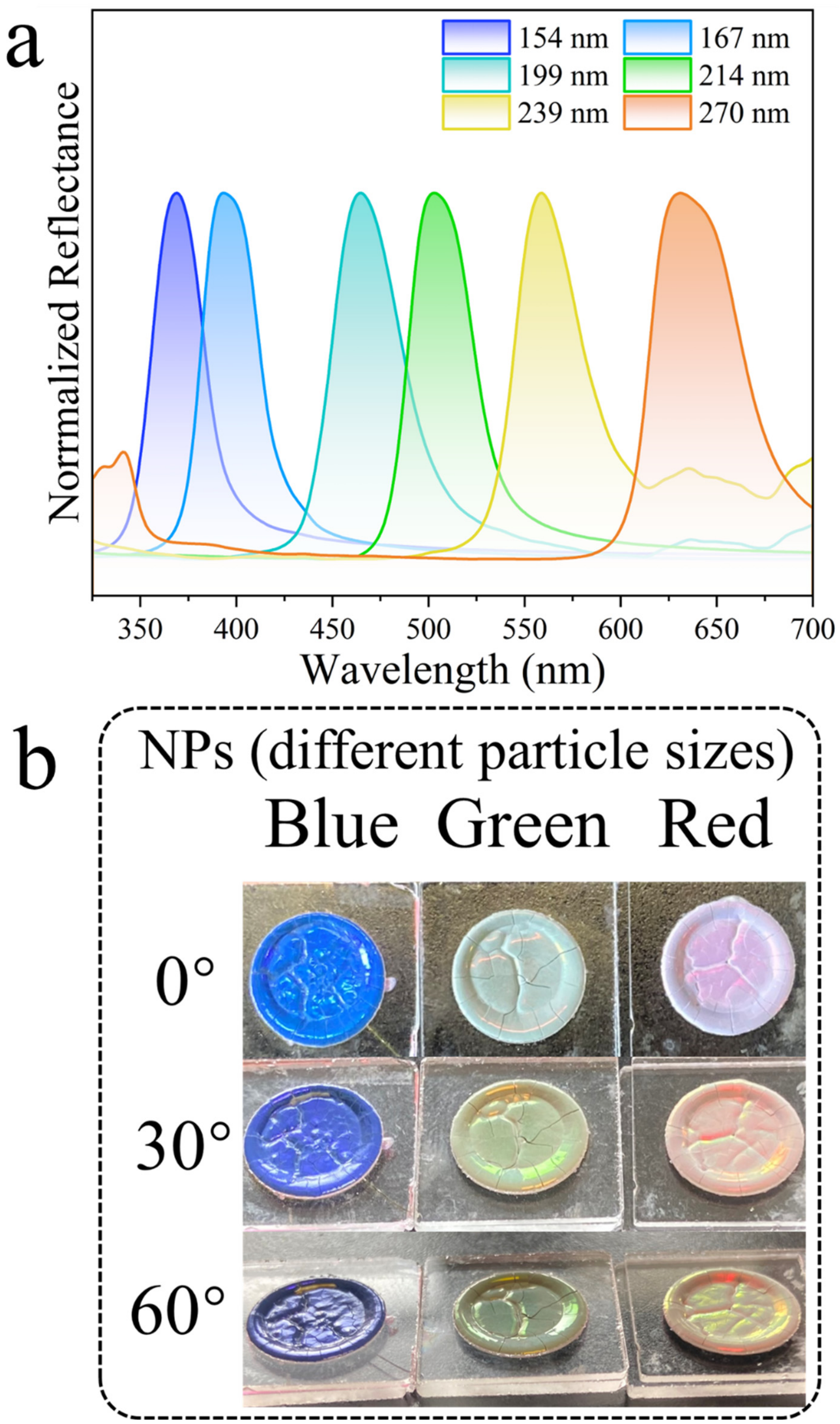

Figure S17. a) Normal-incidence reflection spectra of photonic crystals formed by NPs with varying diameters; b) The angle-dependent structural color of NPs arrays under different viewing angles.

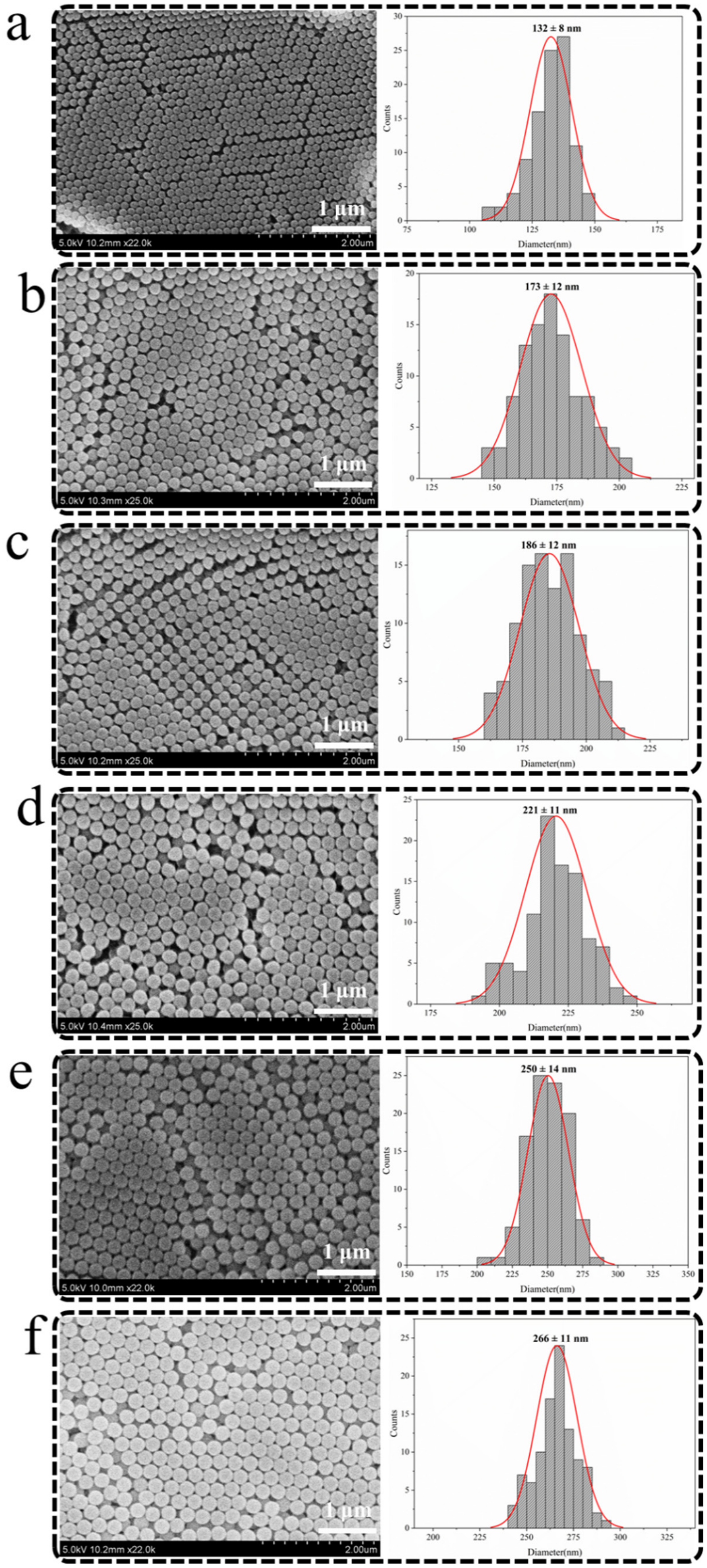

Figure S18. (a-f) SEM images (left panels) and corresponding size distribution histograms (right panels) of nanoparticles (NPs) with controlled size variation. Particle diameters increase progressively from (a) to (f). Scale bars: 1 μm.

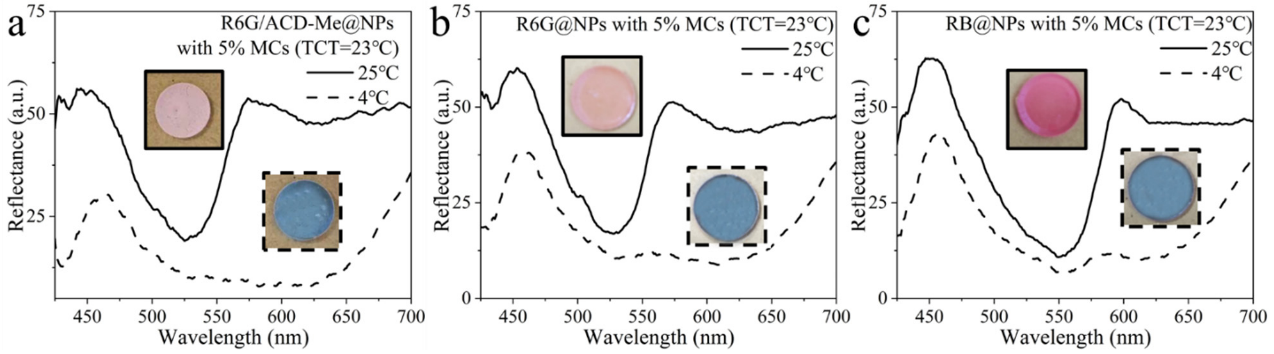

Figure S19. Temperature-dependent reflectance spectra (4 °C vs 25 °C) with corresponding sample photographs (above each curve) for: (a) R6G/ACD-Me@NPs (5% MCs, TCT = 23 °C), (b) R6G@NPs (5% MCs, TCT = 23 °C), and (c) RB@NPs (5% MCs, TCT = 23 °C).

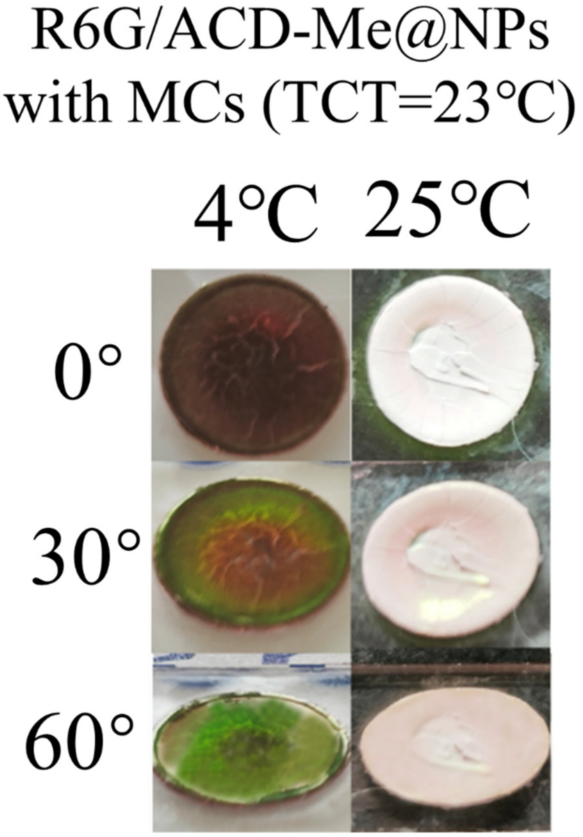

Figure S20 The color of R6G/ACD-Me@NPs with 5% MCs (TCT=23°C) under different viewing angles and temperatures.

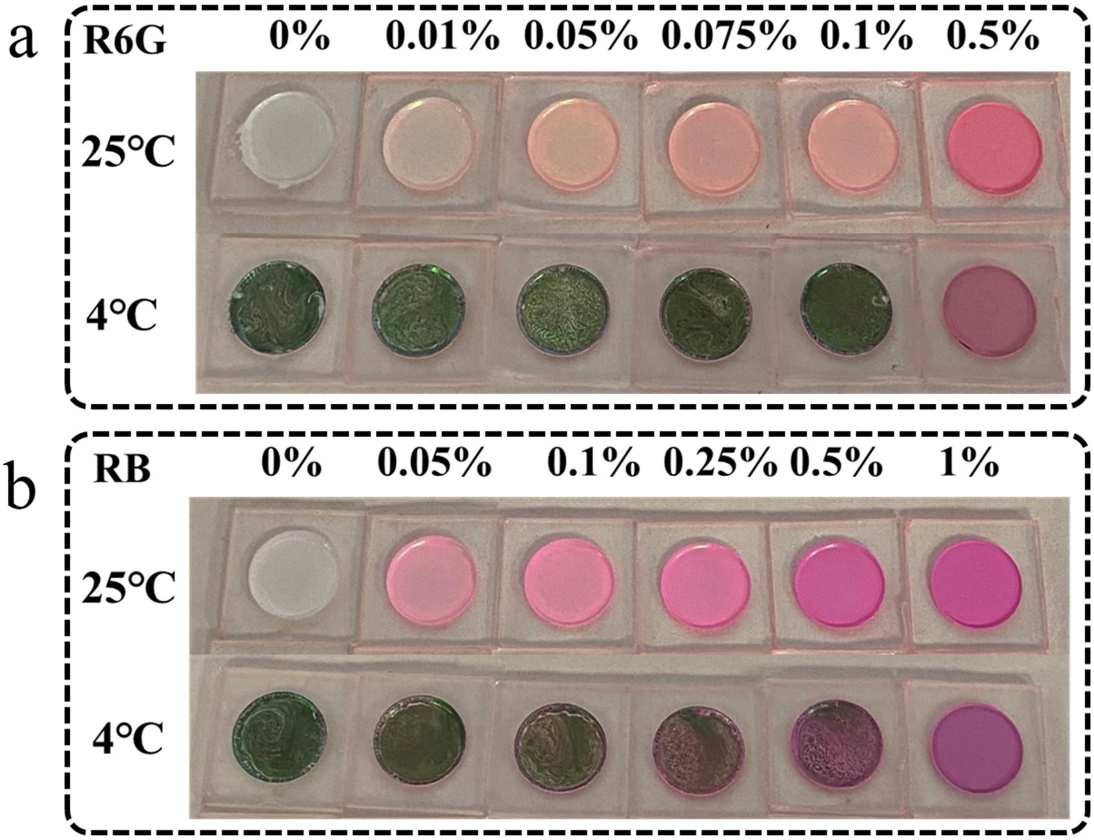

Figure S21. (a) Photographs of R6G/ACD-Me@NPs (5% MCs) with varying R6G concentrations at 4 °C and 25 °C; (b) Photographs of RB/ACD-Me@NPs (5% MCs) with different RB concentrations under the same temperature conditions.

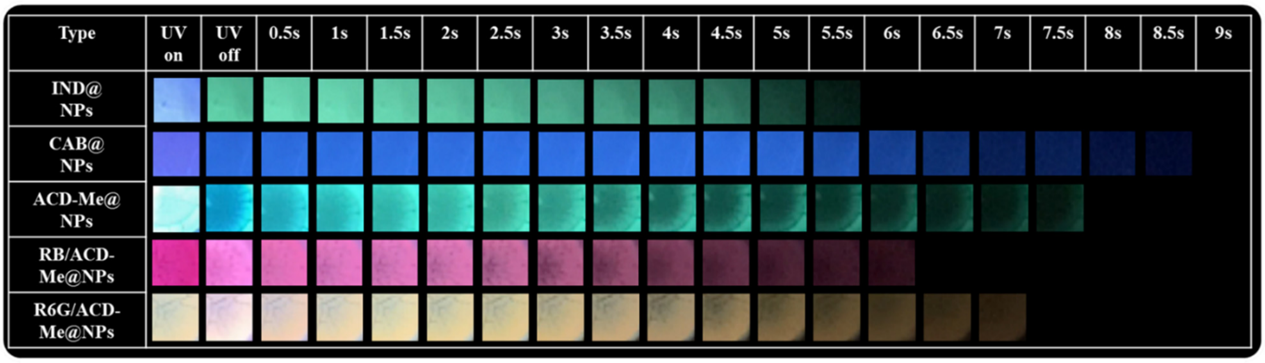

Figure S22. Digital images of IND@NPs, CAB@NPs, ACD-Me@NPs, R6G/ACD-Me@NPs and RB/ACD-Me@NPs with UV radiation on and off, CAB@NPs excitation wavelength: 310 nm, the rest of the excitation wavelengths: 395 nm, R6G/RB doping concentration: 0.075 wt%/0.25 wt%.

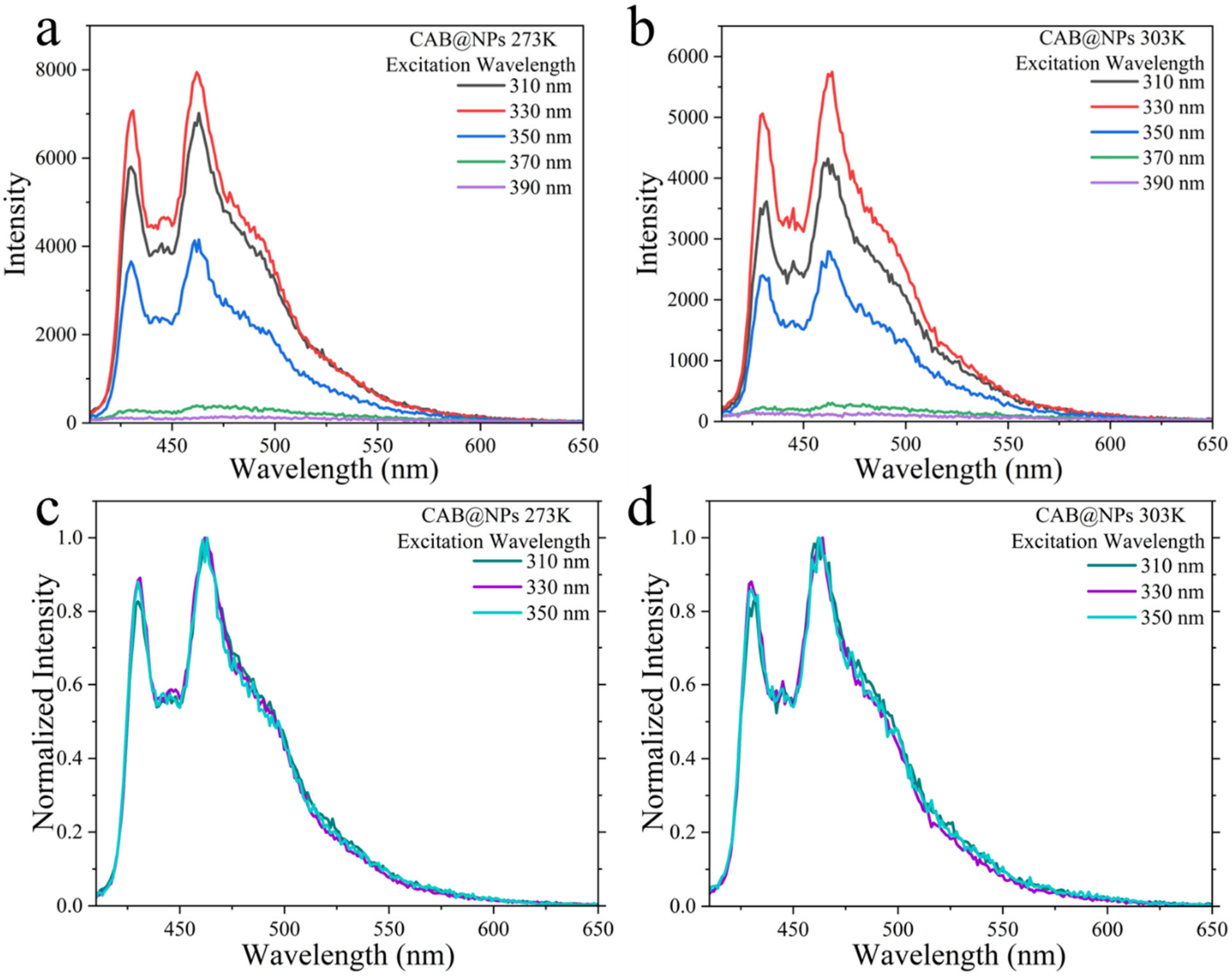

Figure S23. a) Delayed PL spectra of CAB@NPs under different excitation wavelengths at 273 K, b) Delayed PL spectra of CAB@NPs under different excitation wavelengths at 303 K, c) Normalized delayed PL spectra of CAB@NPs under different excitation wavelengths at 273 K, d) Normalized delayed PL spectra of CAB@NPs under different excitation wavelengths at 303 K.

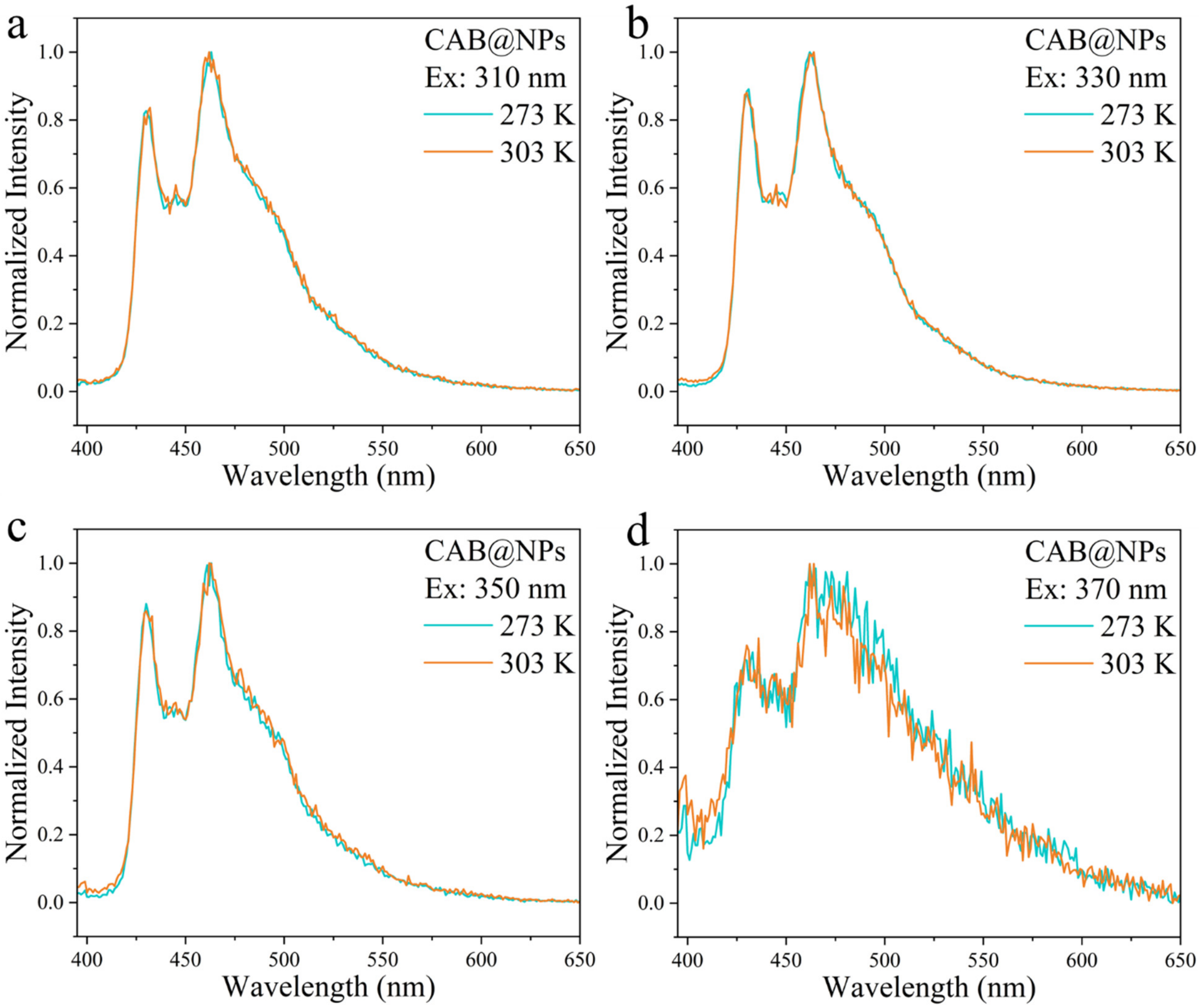

Figure S24. Delayed PL spectra of CAB@NPs at 273 K and 303 K under a) 310 nm; b) 330 nm; c) 350 nm and d) 370 nm excitation wavelengths.

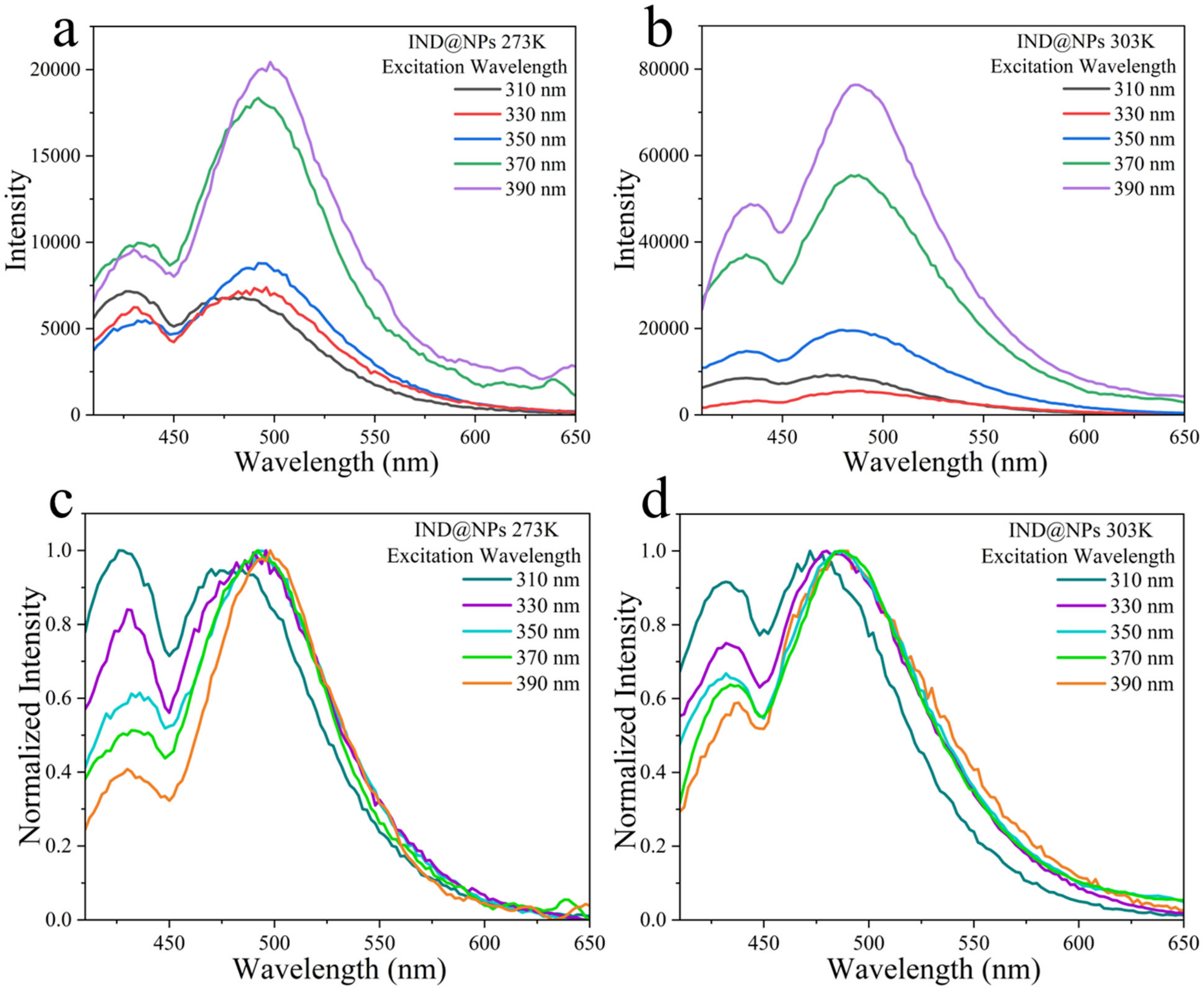

Figure S25. a) Delayed PL spectra of IND@NPs under different excitation wavelengths at 273 K, b) Delayed PL spectra of IND@NPs under different excitation wavelengths at 303 K, c) Normalized delayed PL spectra of IND@NPs under different excitation wavelengths at 273 K, d) Normalized delayed PL spectra of IND@NPs under different excitation wavelengths at 303 K.

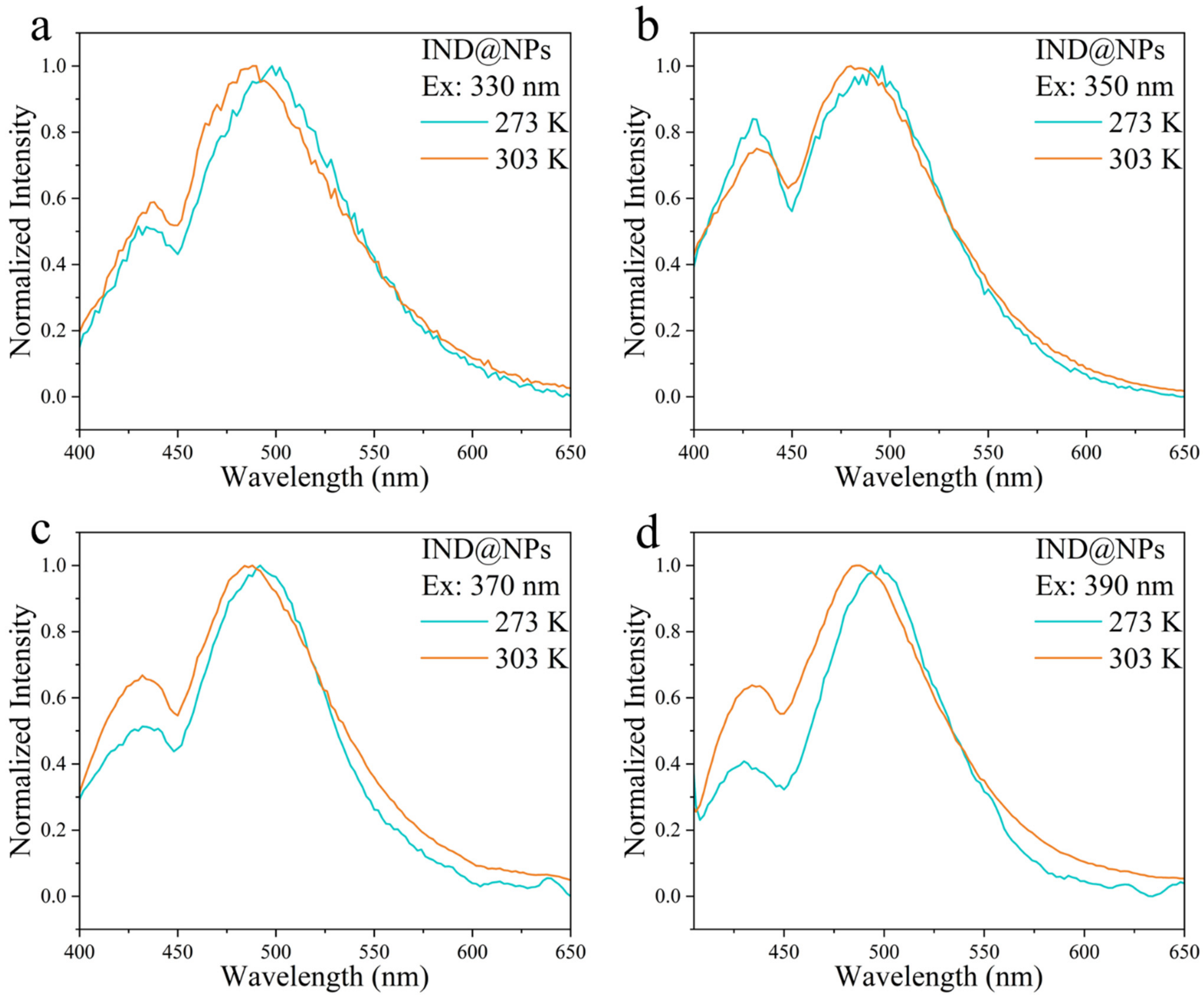

Figure S26. Delayed PL spectra of IND@NPs at 273 K and 303 K under a) 330 nm; b) 350 nm; c) 370 nm and d) 390 nm excitation wavelengths.

2.2 Supplementary tables

Table S1 Energy levels, transition configurations and spin-orbit coupling constants of the singlet and triplet excited states of ACD-Me.

Excited State	Energy Level (Wavelength)	Transition Configuration	SOC Constant (cm^{-1})
S_1	3.26 eV (380 nm)	H->L 97%	
T_1	2.35 eV (528 nm)	H-2->L 6%<br>H->L 92%	S_0 0.3214<br>S_1 0.0131
T_2	2.72 eV (456 nm)	H-1->L 96%<br>H-1->L+6 3%	S_0 40.0194<br>S_1 13.5825
T_3	3.17 eV (391 nm)	H-4->L+2 7%<br>H-3->L+1 4%<br>H-2->L 77%<br>H->L 6%<br>H->L+3 3%	S_0 3.5038<br>S_1 1.0624
T_4	3.29 eV (377 nm)	H-4->L 6%<br>H-4->L+3 4%<br>H-3->L 40%<br>H-2->L+2 8%<br>H->L+1 36%<br>H->L+2 3%	S_0 0.1858<br>S_1 0.1296
T_5	3.74 eV (332 nm)	H-4->L 45%<br>H-2->L+2 4%<br>H->L+1 32%<br>H->L+2 17%	S_0 1.1710<br>S_1 0.4442
T_6	3.85 eV (322 nm)	H-4->L 3%<br>H-3->L 47%<br>H->L+1 24%<br>H->L+2 23%	S_0 1.5362<br>S_1 0.2900
T_7	4.25 eV (292 nm)	H-4->L+1 14%<br>H-4->L+2 6%<br>H-3->L+1 18%<br>H-2->L 10%<br>H->L+3 47%	S_0 0.1624<br>S_1 0.3282

Table S2 Simulated average scattering free paths in assembled NPs (refractive index of air = 1)

NPs radius (nm)	Incident wavelength (nm)	Refractive index of NPs	Average scattering free path (nm)
100	425	1.4969	147.7
100	450	1.4967	146.7
100	470	1.4965	145.9
100	560	1.4950	143.4
100	580	1.4947	143.0
100	600	1.4944	142.6

Table S3 Simulated average scattering free paths in assembled with MCs (refractive index of air = 1, NPs content = 0.95, MCs content = 0.05)

NPs radius (nm)	MCs radius (nm)	Incident wavelength (nm)	Refractive index of NPs	Average scattering free path (nm)
100	2000	425	1.4969	65.0
100	2000	450	1.4967	64.5
100	2000	470	1.4965	64.2
100	2000	560	1.4950	63.1
100	2000	580	1.4947	62.9
100	2000	600	1.4944	62.7

2.3 Supplementary MATLAB code for average scattering free path simulation

2.3.1 Theoretical calculation code for the average scattering free path of pure NPs photonic crystals

```
function mean_free_path = calculate_mean_free_path ()
% Input parameters
   = input (' Please enter the scatterer radius (unit:) nm): '); % Scattering rad
ius (nm)
   = input (' Please enter the wavelength of the incident light (unit: nm): '); %
 Incident light wavelength (nm)
  = input (' Please enter the refractive index of the scatterer: '); % Scattering
body refractive index
```

```
  = input (' Please input the refractive index of the background medium: '); %
 Background medium refractive index
  = input (' Please enter the scattering center density (unit:) m^-3): '); % Sca
ttering center density (m ^ -3)

% Unit conversion (nm -> m)
a = a * 1e-9; % Convert to meters
lambda = lambda * 1e-9; % Convert to meters

% Calculate the size parameter x
x = 2 * pi * a * nm / lambda;

% Calculate scattering efficiency Q_stca (using Mie scattering theory)
Q_sca = calculate_mie_scattering_efficiency (x, np, nm);

% Calculate the scattering cross-section sigma
sigma = Q_sca * pi * a^2;

% Calculate the average scattering free path
mean_free_path = 1 / (rho * sigma);

% Output result
Fprintf (' Mean Scattering Free Path: %.4f μm\n', mean_free_path * 1e6);
end

function Q_sca = calculate_mie_scattering_efficiency (x, np, nm)
% Calculate Mie scattering efficiency Q_stca
% Simplified formula using Mie scattering theory (applicable to small-sized
parameter x)
% For more accurate calculations, the complete Mie scattering coefficient ca
n be used

m = np / nm; % Refractive Index Comparison
Q_sca = 2; % Initial assumption (for small particles, Q_stca is close to 2)

% If the size parameter x is large, more complex Mie scattering calculations
can be used
if   > 0.1
% Use the miepython library or customize Mie scattering coefficient calculat
ion
% Here, a simplified formula is used
Q_sca = 2 + 4 * (m^2 – 1) ^2 / (m^2 + 2) ^2 * sin(x)^2 / x^2;
end
```

```
end
```

2.3.2 Theoretical calculation code for the average scattering free path of NPs photonic crystals with microcapsules

```
function=calculate_mean_free_path_two_scatterers_with_volume_fraction ()
   % Input parameters
        = input (' Please enter the microsphere radius (unit:) nm): '); % Microsphere radius (nm)
       = input (' Please enter the microcapsule radius (unit:) nm): '); % Microcapsule radius (nm)
      = input (' Please enter the wavelength of the incident light (unit: nm): '); % Incident light wavelength (nm)
      = input (' Please enter microsphere refractive index: '); % Microsphere refractive index
      = input (' Please enter microcapsule refractive index: '); % Microcapsule refractive index
      = input (' Please input the refractive index of the background medium: '); % Background medium refractive index
      = input (' Please enter the volume fraction of microspheres:'); % Microsphere volume fraction
      = input (' Please enter the volume fraction of microcapsules: '); % Microcapsule volume fraction

   % Unit conversion (nm -> m)
   a1 = a1 * 1e-9; % Convert to meters
   a2 = a2 * 1e-9; % Convert to meters
   lambda = lambda * 1e-9; % Convert to meters

   % Calculate the density of microspheres and microcapsules
   rho1 = f1 / (4/3 * pi * a1^3); % Microsphere density
   rho2 = f2 / (4/3 * pi * a2^3); % Microcapsule density

   % Calculate the scattering cross-section sigma1 of microspheres
   x1 = 2 * pi * a1 * nm / lambda; % Microsphere size parameters
   Q_sca1 = calculate_mie_scattering_efficiency (x1, np1, nm); % Microsphere scattering efficiency
   sigma1 = Q_sca1 * pi * a1^2; % Microsphere scattering cross-section

   % Calculate the scattering cross-section sigma2 of microcapsules
   x2 = 2 * pi * a2 * nm / lambda; % Microcapsule size parameters
   Q_sca2 = calculate_mie_scattering_efficiency (x2, np2, nm); % Scattering efficiency of microcapsules
```

```
    sigma2 = Q_sca2 * pi * a2^2; % Microcapsule scattering cross-section

    % Calculate the total scattering center density rho
    rho = rho1 + rho2;

    % Calculate the average scattering cross section<sigma>
    mean_sigma = (rho1 * sigma1 + rho2 * sigma2) / rho;

    % Calculate the average scattering free path
    mean_free_path = 1 / (rho * mean_sigma);

    % Output result
    Fprintf (' Mean Scattering Free Path: %.4f μm\n', mean_free_path * 1e6);
end

function Q_sca = calculate_mie_scattering_efficiency (x, np, nm)
    % Calculate Mie scattering efficiency Q_stca
    m = np / nm; % Refractive Index Comparison
    Q_sca = 2 + 4 * (m^2 - 1) ^2 / (m^2 + 2) ^2 * sin(x)^2 / x^2; % Simplified
formula
End
```

3. References

[1] a) Geoffroy J. Aubry, Lukas Schertel, Mengdi Chen, Henrik Weyer, Christof M. Aegerter, Sebastian Polarz, Helmut Cölfen, G. Maret, Phys. Rev. A 2017, 4, 43871; b) L. Schertel, L. Siedentop, J. M. Meijer, P. Keim, C. M. Aegerter, G. J. Aubry, G. Maret, Adv. Optical. Mater. 2019, 7, 1900442.

[2] M. J. Frisch, G. W. Trucks, H. B. Schlegel, G. E. Scuseria, M. A. Robb, J. R. Cheeseman, G. Scalmani, V. Barone, G. A. Petersson, H. Nakatsuji, X. Li, M. Caricato, A. V. Marenich, J. Bloino, B. G. Janesko, R. Gomperts, B. Mennucci, H. P. Hratchian, J. V. Ortiz, A. F. Izmaylov, J. L. Sonnenberg, Williams, F. Ding, F. Lipparini, F. Egidi, J. Goings, B. Peng, A. Petrone, T. Henderson, D. Ranasinghe, V. G. Zakrzewski, J. Gao, N. Rega, G. Zheng, W. Liang, M. Hada, M. Ehara, K. Toyota, R. Fukuda, J. Hasegawa, M. Ishida, T. Nakajima, Y. Honda, O. Kitao, H. Nakai, T. Vreven, K. Throssell, J. A. Montgomery Jr., J. E. Peralta, F. Ogliaro, M. J. Bearpark, J. J. Heyd, E. N. Brothers, K. N. Kudin, V. N. Staroverov, T. A. Keith, R. Kobayashi, J. Normand, K. Raghavachari, A. P. Rendell, J. C. Burant, S. S. Iyengar, J. Tomasi, M. Cossi, J. M. Millam, M. Klene, C. Adamo, R. Cammi, J. W. Ochterski, R. L. Martin, K. Morokuma, O. Farkas, J. B. Foresman, D. J. Fox, Wallingford, CT Gaussian 16 Rev. C.01 2016.

[3] X. Gao, S. Bai, D. Fazzi, T. Niehaus, M. Barbatti, W. Thiel, J. Chem. Theory Comput. 2017, 13, 515-524.

[4] S. G. Chiodo, N. Russo, J. Comput. Chem. 2009, 30, 832-839.

[5] Akira Ishimaru. 1978, Wave propagation and scattering in random media, Academic Press, New York, ISBN: 77074051

[6] H. C. van de Hulst. 1957, Light scattering by small particles, Structure of matter series, Wiley, New York, ISBN: 57005936

[7] Donald R. Huffman and Craig F. 1983, Bohren, Absorption and scattering of light by small particles, Wiley, New York, ISBN: 82020312

[8] Milton. Kerker. 1969, The scattering of light, and other electromagnetic radiation., Academic Press, New York, ISBN: 68026644

[9] Jin Au Kong, Robert T. Shin and Leung. 1985, Tsang, Theory of microwave remote sensing, Wiley series in remote sensing, Wiley, New York, ISBN: 84017397

[10] Michael I. Mischenko. 2002. Scattering, Absorption, and Emission of Light by Small Particles, Cambridge University Pres, Cambridge, ISBN: 9780521782524
